# Task-Dependent Changes in Cross-Level Coupling between Single Neurons and Oscillatory Activity in Multiscale Networks

**DOI:** 10.1371/journal.pcbi.1002809

**Published:** 2012-12-20

**Authors:** Ryan T. Canolty, Karunesh Ganguly, Jose M. Carmena

**Affiliations:** 1 Electrical Engineering and Computer Sciences, University of California, Berkeley, Berkeley, California, United States of America; 2 Helen Wills Neuroscience Institute, University of California, Berkeley, Berkeley, California, United States of America; 3 San Francisco VA Medical Center, San Francisco, California, United States of America; 4 Neurology, University of California, San Francisco, San Francisco, California, United States of America; 5 UCB/UCSF Joint Graduate Group in Bioengineering, University of California, Berkeley, Berkeley, California, United States of America; Indiana University, United States of America

## Abstract

Understanding the principles governing the dynamic coordination of functional brain networks remains an important unmet goal within neuroscience. How do distributed ensembles of neurons transiently coordinate their activity across a variety of spatial and temporal scales? While a complete mechanistic account of this process remains elusive, evidence suggests that neuronal oscillations may play a key role in this process, with different rhythms influencing both local computation and long-range communication. To investigate this question, we recorded multiple single unit and local field potential (LFP) activity from microelectrode arrays implanted bilaterally in macaque motor areas. Monkeys performed a delayed center-out reach task either manually using their natural arm (Manual Control, MC) or under direct neural control through a brain-machine interface (Brain Control, BC). In accord with prior work, we found that the spiking activity of individual neurons is coupled to multiple aspects of the ongoing motor beta rhythm (10–45 Hz) during both MC and BC, with neurons exhibiting a diversity of coupling preferences. However, here we show that for identified single neurons, this beta-to-rate mapping can change in a reversible and task-dependent way. For example, as beta power increases, a given neuron may increase spiking during MC but decrease spiking during BC, or exhibit a reversible shift in the preferred phase of firing. The within-task stability of coupling, combined with the reversible cross-task changes in coupling, suggest that task-dependent changes in the beta-to-rate mapping play a role in the transient functional reorganization of neural ensembles. We characterize the range of task-dependent changes in the mapping from beta amplitude, phase, and inter-hemispheric phase differences to the spike rates of an ensemble of simultaneously-recorded neurons, and discuss the potential implications that dynamic remapping from oscillatory activity to spike rate and timing may hold for models of computation and communication in distributed functional brain networks.

## Author Summary

How is the functional role of a particular neuron established within an ensemble? The concept of a neural tuning curve – the mapping from input variables such as movement direction to output firing rate – has proven useful in investigating neural function. However, prior work shows that tuning curves are not fixed but may be remapped as a function of task demands – presumably via high-level mechanisms of cognitive control. How is this accomplished? Brain rhythms may play a causal role in this process, but the coupling of single cells to network activity remains poorly understood. We investigated the coupling between rhythmic beta activity and spiking as macaques performed two different tasks. This coupling can be described in terms of a function that maps oscillatory amplitude and phase to instantaneous spike rate. Similarly to direction tuning, this “internal” tuning curve also exhibits task-dependent changes. We characterize these changes across a large ensemble of simultaneously-recorded cells, and consider some of the neuro-computational implications presented by cross-level coupling between single cells and large-scale networks. In particular, relative to the slow time-scale of behavior, the observed beta-to-rate mappings may prove useful for modulating winner-take-all dynamics on intermediate time-scales and relative spike timing on fast time-scales.

## Introduction

Our understanding of the biophysical mechanisms governing the dynamics of single neurons has increased dramatically over the past decades. In contrast, a principled understanding of the mechanisms governing ensembles of interacting neurons – from local cortical microcircuits to discrete functional areas to large-scale brain networks – remains elusive. Recent imaging advances have generated detailed structural maps that span from the micro-scale of local synaptic connectivity [1]–[3] to the macro-scale of hierarchical, long-range cortical networks [4]–[6]. However, a comprehensive description of the brain connectome [5] – the structural connectivity of the nervous system – is a necessary but not sufficient condition for understanding the dynamic coordination of brain networks. To survive in a complex world, agents must switch quickly from one task to another – for example, switching from thinking about an article to dodging a speeding car when crossing an intersection. Different tasks require the differential activation of separate functional networks, including (but not limited to) changes in the mean activity of multiple brain areas (nodes) as well as transient modulation of the effective strength of connectivity between areas (directed links). Importantly, the modulation of distinct networks required for task switching occurs fast enough that structural connectivity can be considered relatively fixed. How is this dynamic coordination of networks accomplished?

Several groups have proposed that neuronal oscillations play a critical role in the dynamic coordination of multi-scale brain networks [7]–[22]. In this view, oscillations or brain rhythms may influence both local cortical computation [7], [8] as well as long-range communication [21]. Furthermore, Lakatos et al. [17] proposed that a hierarchy of interacting oscillations, where low frequency phase modulates high frequency power, may serve to coordinate information flow across multiple spatial and temporal scales [18]. Most hypotheses about how oscillations may influence neural coding and network coordination predict that the spiking of single neurons is statistically dependent upon one or more distinct frequency bands of the local field potential (LFP). We refer to this statistical dependence between the micro-scale of single neurons and the meso- and macro-scale of oscillatory network activity as cross-level coupling (CLC). Importantly, if neuronal oscillations play a role in coordinating distributed networks, then we would expect to observe CLC between the neurons embedded in those networks and the different brain rhythms associated with their functional activation. That is, failure to observe CLC would be evidence against oscillations playing a role in dynamic coordination. Conversely, if CLC is present, then a quantitative descriptive model of the CLC observed within neuronal ensembles across different tasks may help distinguish between different mechanistic accounts of how brain rhythms modulate activity in distributed functional networks. That is, characterizing the distribution and stability of CLC parameters across neurons and tasks is an essential step that may prove useful for selecting between different possible mechanisms of oscillatory network control.

CLC between spikes and internally-generated brain rhythms remains less well understood than the coupling between spikes and externally-associated factors such as visual orientation [23], [24] or movement direction [25], [26]. Nonetheless, empirical evidence for CLC between single neurons and a variety of different brain rhythms has been observed in several brain areas across a number of behavioral tasks [22], [27]–[41]. This evidence includes spike-field coherence between single neurons and the gamma (30–80 Hz) rhythm in the hippocampus [33], [37], basal ganglia [42], and a variety of different cortical areas [22],[29],[43]. CLC in the theta (4–8 Hz) band has been observed in the hippocampus [34] and between the hippocampus and prefrontal cortex [38], with theta-phase precession of hippocampal place cells [44] serving as a prototype of dynamic CLC. Event-related changes in the power and synchronization of the gamma and theta bands suggests that these rhythms are related to functional activation [45]. For example, gamma synchronization in visual cortex is modulated by attention [39] and predicts the speed of change detection [46], while increased hippocampal theta power precedes successful memory encoding in humans [47]. Furthermore, the role of oscillatory phase (distinct from oscillatory power) in neuronal coding and communication has emerged as a topic of growing interest. It was recently shown that spikes from prefrontal neurons occurring at particular phases of 32-Hz filtered LFPs were more informative about object coding during a working memory task than were spikes occurring at other phases [22]. Similarly, posterior parietal neurons coherent with 15-Hz beta activity were predictive of reaction times during a coordinated reach-saccade task, while other actively spiking neurons were not [32]. In primary visual cortex, gamma phase modulates orientation selectivity and noise correlations [43], while entrainment of delta (1–4 Hz) phase in visual cortex increased response gain and speeded reaction times [48].

However, compared to other rhythms such as theta and gamma, the cellular/network origins [49], [50] and functional role [51]–[56] of sensorimotor beta oscillations are less well understood and remain subjects of considerable debate [16]. In primary motor cortex, Murthy and Fetz [27] were among the first to show that spike timing is dependent on the phase of the motor beta (10–40 Hz) rhythm, with stronger phase-locking occurring when beta power is high. Reimer and Hatsopoulos [57] showed that precise spike timing depends on the combined influence of both external events as well as internally-generated ongoing beta activity – that is, motor cells are tuned to internal as well as external events. Intriguingly, beta activity is a mesoscopic phenomenon arising from microscale network interactions and results in propagating spatiotemporal waves that can encode information about upcoming movements [58], [59] and action goals [60]. The strong coupling between single cells and the beta rhythm, on the one hand, and between beta and experimental task demands [52], [61], [62], on the other, suggests that beta-band CLC in the motor system may prove useful in understanding the connection of single cells to the dynamic activation of functionally-defined neuronal ensembles.

**Figure 1:**
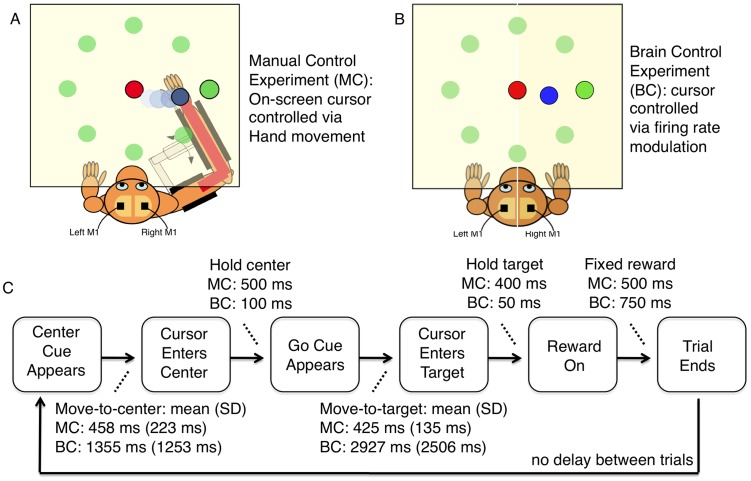
The Manual Control (MC) and Brain Control (BC) tasks. A) Schematic of the MC task, where monkeys use their right arm to perform a delayed center-out reaching task to move an on-screen cursor from a center cue to one of 8 peripheral targets. B) Schematic of the BC task, where monkeys use changes in the firing rates of a subset of recorded cells in order to move a cursor from the center to one of 8 targets (irrespective of physical movement). C) Timing of different trial sub-stages in the MC and BC tasks. Trials start with the appearance of central cue. A hold period (MC: 500 ms; BC: 100 ms) begins once the cursor enters the central cue. Upcoming target appears onscreen once cursor enters center. Go cue (central cue color change) indicates that monkeys can move the cursor to the designated target (via hand movement in MC or firing rate changes in BC), with a range of movement times. Holding the cursor within the target (MC: 400 ms; BC: 50 ms) triggers juice reward (500 ms), followed by the start of the next trial.

Therefore, a primary purpose of this study was to provide a quantitative characterization of CLC between the motor beta rhythm (10–45 Hz) and a large ensemble of simultaneously-recorded neurons in primary motor cortex (M1) across two distinct but related behavioral tasks (Figure 1). Such a characterization of CLC is required both for understanding the functional role of the beta rhythm in the motor system, on the one hand, as well as for evaluating the hypothesized role oscillations may play in coordinating large-scale networks more generally. Importantly, several aspects of CLC remain unclear. First, the degree of heterogeneity of CLC parameters across a population remains uncertain. Many prior studies employed acute recordings of single cells or small ensembles of simultaneously-recorded cells, pooling cells recorded serially over different days in order to make inferences about the distribution of CLC parameters over the neuronal population. This ergodic assumption makes it difficult to distinguish the case where a wide range of CLC parameters holds in a stable fashion for an ensemble over time, on the one hand, from the case where each cell is described by similar CLC parameters, but those parameters evolve dynamically over time. An advantage of the chronically-implanted microelectrode arrays used here is that a large ensemble of neurons can be recorded simultaneously, with identified single units followed over several days, permitting an unbiased assessment of the population diversity of CLC that holds during a given task. Second, it remains unclear how stable within-neuron CLC parameters are across different tasks. Few studies have investigated CLC in a given neuron over different tasks – that is, it remains unclear how variable CLC is within a given neuron as the subject switches from one task to another. This study examines CLC within the same neuron for two different tasks that have similar high-level goals, but are associated with distinct activation patterns – namely, a delayed center-out movement task performed via motion of the hand (Manual Control, MC) or via modulation of neuron firing rates (Brain Control, BC; see Materials and Methods). Third, the relative importance of different aspects of oscillatory activity remain unclear. CLC has most often been assessed using spike- or cycle-triggered averages, or via spike-field coherence, but both of these methods combine oscillatory amplitude and oscillatory phase into one measure. Furthermore, these measures do not assess the dependence of spiking upon other factors such as synchronization between different regions. By computing these dependencies for the same set of neurons across conditions, the relative importance of different oscillatory factors to a given neuron can be made clear. Thus, characterizing the CLC of a large population of simultaneously-recorded neurons over two different tasks enables us to describe both the within-task, cross-neuron diversity of coupling to different aspects of oscillatory activity, as well as the cross-task, within-neuron stability (or dynamic remapping) of coupling that may occur between spiking and beta activity in the motor cortex.

Here we present several findings. First, we provide a descriptive (phenomenological) model of the coupling between the instantaneous spike rate of a given cell and frequency-specific oscillatory activity. Importantly, this model accounts separately for the influence on rate of oscillatory amplitude, phase, and the interaction between amplitude and phase. Second, we show that this model describes the coupling for a large ensemble of cells, but that a wide range of model parameters holds across the population during a given task. In particular, some cells were more sensitive to amplitude than phase or vice versa, or had differential sensitivity to the interaction between amplitude and phase. Third, for a given cell the coupling of beta activity to spiking was stable across multiple sessions of a given task, but was often remapped when subjects switched to a different task. The parameter changes induced across the ensemble by this reversible remapping were reliable across multiple datasets. Fourth, it appears that this rhythm-to-rate mapping and task-dependent remapping have properties that would prove useful for the causal control of functional networks interactions. We conclude with a discussion of how these empirical results point to potential mechanisms for the control of neuro-computational processes. In sum, cross-level coupling between micro-scale spiking and meso- and macro-scale network activity appears to be a robust, flexible bridge linking together the different levels of brain organization required for effective perception, cognition, and action.

## Materials and Methods

### Surgery and electrophysiology

Two adult male rhesus monkeys (*Macaca mulatta*) were chronically implanted with multiple microelectrode arrays. Each array consisted of 64 Teflon-coated tungsten microelectrodes (35 mm in diameter, 500-mm interelectrode spacing) arranged in an 8 × 8 array designed to target cortical layer V (Innovative Neurophysiology, Durham, NC). Monkey P was implanted bilaterally in the arm area of primary motor cortex (M1), and in the arm area of left hemisphere dorsal premotor cortex (PMd), for a total of 192 electrodes across 3 implants. 95 identified single units from this monkey were examined. Monkey R had bilateral implants in the arm area of M1 and PMd, for a total of 256 electrodes across four implants. 86 identified single units from this monkey were examined. Localization was performed using stereotactic coordinates [63]. Implants targeted layer-5 pyramidal tract neurons and were positioned at a depth of 3 mm (M1) or 2.5 mm (PMd). Intraoperative monitoring of spike activity guided electrode depth. See [64] for full experimental details. A 128 channel multi-acquisition processor (MAP) system (Plexon Inc., Dallas, TX) was used to record unit activity. Only single units that had a clearly identified waveform with a signal-to-noise ratio of at least 4∶1 were used. An on-line spike-sorting application (Sort-Client; Plexon Inc., Dallas, TX) was used to sort activity prior to recording sessions. Large populations of well-isolated units and up to 128 LFP channels (1 kHz sampling) were recorded during daily sessions for both monkeys. Conducted procedures were in compliance with the National Institutes of Health Guide for the Care and Use of Laboratory Animals and approved by the University of California at Berkeley Institutional Animal Care and Use Committee.

### Behavior

Monkeys were trained to perform a delayed center-out reaching task using either their natural arm inside a Kinarm exoskeleton (BKIN Technologies, Kingston, Ontario) (Manual Control, MC), or under direct neural control through a brain-machine interface (BMI) and irrespective of overt physical movement (Brain Control, BC) [64]. Monkeys self-initiated trials by bringing the cursor to the center for a hold period (MC, 500 ms; BC, 100 ms), followed by the presentation of a GO cue (color change of center cue). A trial error occurred if the cursor failed to reach the target within 10 s after a GO cue. The goal was to perform a center-out task, moving the cursor from the center to one of eight peripheral targets distributed over a 14-cm diameter circle. Required hold times at target were 400 ms for MC and 50 ms for BC. Target radius was typically 0.75 cm. A liquid reward was provided after a successful reach to each target. During training and recording animals sat in a primate chair with their heads restrained. During BC sessions the Kinarm was removed and the arms restrained to the primate chair.

### LFP filtering and event-related analysis

**Figure 2:**
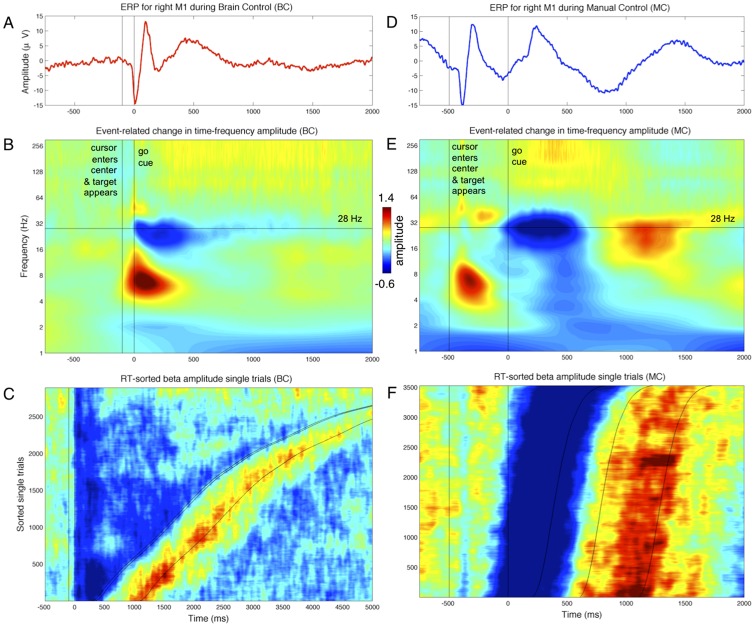
Event-related changes in local field potential (LFP) activity. A) Go-cue related ERP activity in right primary motor cortex (M1) during Brain Control (BC). The neurons driving the brain-machine interface are in right M1. B) Time-frequency plot showing frequency-specific changes in mean amplitude relative to the onset of the go cue (0 ms) in right M1 during BC, including amplitude changes in the theta (6 Hz), high beta (28 Hz), low gamma (36 Hz), and high gamma (>70 Hz) bands. Across all frequencies, color scale indicates increase (red) or decrease (blue) in amplitude relative to the mean of 1. Notice the strong drop in beta amplitude locked to the onset of the go cue. C) Smoothed single-trial traces of beta amplitude, sorted by movement duration (from go cue to cursor entering target). Vertical black line at −100 ms indicates when cursor entered the center cue, 0 ms is go cue onset. First curved black line indicates when cursor enters target, second indicates reward onset, and third indicates reward offset and beginning of the next trial. The sharp drop in beta amplitude during movement is followed by a beta amplitude increase during the reward delivery. D–F) As in A–C, during Manual Control (MC).

Analyses were done using MATLAB (Mathworks). Filtering to extract beta amplitudes and phases was performed by convolving signals with Gabor time-frequency basis functions (Gaussian envelope). A Gabor time-frequency atom is fully defined by three parameters; namely, the center time t_0_, the center frequency v_0_, and the duration parameter s_0_. In the time domain, the Gabor atom g is given as g(t | t_0_, v_0_, s_0_) = 2^1/4^ exp[( − 1/4) s_0_ − p(t − t_0_)^2^ exp[ − s_0_] + 2p v_0_ (t − t_0_)]. Since there was no significant difference in the frequency corresponding to the power spectral peak (power of −46 10*log_10_(µV^2^/Hz) at a frequency of 28 Hz), a fixed center frequency v_0_ of 28 Hz centered on the observed PSD peak and a duration parameter s_0_ of −5.075 (frequency domain standard deviation of 3.57 Hz) were used to extract the “beta signal” this study. For the amplitude-to-rate, phase-to-rate, phase-difference-to-rate, and amplitude-to-weight mappings, a spatial average of all LFPs recorded from the 64 electrodes in one microelectrode array was generated and used as the raw input signal. Each 8 × 8 microelectrode array covers an area of 3.5 × 3.5 mm^2^, and therefore this spatial average is similar in scale to a single ECoG macroelectrode. Two average signals s_L_ and s_R_, were generated for left and right M1, respectively, prior to additional analyses. After concatenating separate recording blocks, the BC dataset had a duration of 410 minutes for monkey P (97 minutes for monkey R), while the MC dataset had a duration of 172 minutes for monkey P (58 minutes for monkey R). To compute event-related potentials (ERPs) and event-related time-frequency amplitude maps, the signal indices of go cue onsets were identified for BC and MC. For ERPs, trial epochs −1000 ms before to 10000 ms after go cue indices were extracted from signals s_L_ and s_R_ were averaged. For time-frequency analyses, first the signals s_L_ and s_R_ were filtered around a given center frequency as described above. 40 center frequencies spaced semi-logarithmically from 1 to 300 Hz were employed. Second, the amplitude of each filtered signal was normalized such that the mean amplitude across all data was 1. Third, trial epochs −1000 ms before to 10000 ms after go cue indices were extracted from the amplitude time series and averaged. RT-sorted single-trial analyses (e.g., Figure 2C,F) were performed similarly, but rather than averaging all trial epochs together, a sliding window of 250 trials was used after sorting all trials by movement duration. Event-related PSTHs were computed similarly to ERPs, using a binary time series representing spike times.

### Beta amplitude-to-rate, phase-to-rate, and phase-difference-to-rate mappings

To generate the beta amplitude-to-rate mapping, first the time series of instantaneous amplitudes was extracted from one of the average signals (s_L_ or s_R_) described above. Call this N × 1 vector of amplitudes x_A_. Amplitudes were normalized such that the mean amplitude across all data was 1. Second, the spike times from one neuron were used to generate a N × 1 binary vector x_S_, where x_S_[t] equals 1 for spike times t and equals 0 otherwise. Third, a N × 2 matrix M_1_ = [x_A_ x_S_] was formed. Fourth, this matrix M_1_ was truncated to allow reshaping of the array in a future step – given the number of amplitude bins to be used later (n_b_), this matrix M_1_ was truncated to form the N_t_ × 2 matrix M_2_, where N_t_ is the largest integer less than or equal to N for which n_b_ * P = N_t_ for some integer P. Fifth, the rows of the matrix M_2_ were sorted according to the amplitude values in the first column of M_2_; M_2_ = sortrows(M_2_,1). Sixth, a 3-dimensional array of size P × n_b_ × 2 was created by reshaping the sorted matrix M_2_: M_3_ = reshape(M_2_,[P n_b_ 2]). Seventh, the mean amplitude for each bin was computed: A = mean(M_3_(:,:,1),1), where A is a 1 × n_b_ vector of amplitudes. Eighth, the average spike rate for each bin was computed: R = (S_R_/P)*sum(M_3_(:,:,2) =  = 1), where S_R_ is the sampling rate and R is a 1 × n_b_ vector of spike rates. The rate values R over the amplitude support A describe the empirically-observed amplitude-to-rate mapping. Ninth, this histogram-based mapping was fit with 4-parameter sigmoidal function F_S_ using the MATLAB function lsqcurvefit.m, where F_S_(a) = p_1_ + p_2_ tanh ((a − p_3_)/(2 p_4_)), where a<0 is beta amplitude and tanh is the hyperbolic tangent function. In order to assess task-related changes, amplitude-to-rate mappings were computed separately for the full BC and MC datasets. In order to assess within-task stability of the mappings, the BC dataset was split into two disjoint datasets, BC1 and BC2 consisting of odd and even trials, respectively, and the above procedure performed separately on each. Similarly, split-half reliability during MC was assessed using two disjoint datasets MC1 and MC2. The empirical-observed estimates of the beta phase-to-rate and phase-difference-to-rate mappings were produced in an identical way, where the x_A_ time series of step 1 was replaced with a N × 1 vector of instantaneous phases from one hemisphere (phase-to-rate) or a N × 1 vector of inter-hemispheric phase differences (phase-difference-to-rate). For the phase-to-rate mapping, a cosine-type function was used in fitting: F_C_(q) = p_1_ + p_2_ cos (θ − p_3_), where θ is beta phase, p_2_>0, and q, p_3_ are in the interval [ − p, p). For the phase-difference-to-rate mapping, a von Mises-type function was used in fitting: F_D_(j) = p_1_ + p_2_ exp[p_3_ cos (φ − p_4_)], where φ is the phase difference, p_3_>0, and φ, p_4_ is in the interval [ − p, p). For target-specific and trial-stage-specific analyses, the data was presorted to extract only relevant time intervals.

### Amplitude-to-weight mapping

To determine the amplitude-to-weight mapping that describes the multiplicative gain effect beta amplitude has on the phase-to-rate mapping, a procedure similar to that describe above was performed, but sorting datapoints jointly by amplitude and phase. That is, first the amplitude time series x_A_, the phase time series x_P_, and the spike time series x_S_ were combined into a matrix W = [x_A_ x_P_ x_S_], such that each row represents the amplitude, phase, and spike status of one sample point. Second, the rows of W were sorted according to the values in the amplitude column and partitioned into n_ab_ (amplitude) bins, where each bin has the same number of sample points. Third, the data in each (amplitude) bin was further sorted into n_pb_ (phase) bins. Fourth, the spike rate for each (amplitude, phase) bin was computed, generating a n_ab_ × n_pb_ matrix of spike rates. Fifth, this matrix was used to constrain the fitting of the 7-parameter function describing the full beta-to-rate mapping: F_B_(a,θ) = R_BETA_(a,θ) = p_1_ + p_2_ tanh ((a − p_3_)/(2 p_4_)) + (p_5_ a + p_6_ a^2^) cos (θ − p_7_). Given the amplitude-to-rate mapping R_AMP_(a) and the phase-to-rate mapping R_PHASE_(θ) described above, the quadratic weight factor or amplitude-to-rate mapping w_AMP_(a) = b_1_ a + b_2_ a^2^ can be extracted from the relation R_BETA_(a,θ) = R_AMP_(a) + w_AMP_(a) R_PHASE_(θ).

### Multivariate model fitting and predicted rates

The modeling approach described above is inherently univariate and does not extend easily to multivariate approaches. For the multivariate analysis we used a procedure similar to that described in [41], but adapted to account for both amplitudes and phases. First, the N_channel_ LFP signals from a training dataset were filtered to generate a N_channel_ × N_samples_ complex-valued matrix, where for each entry the absolute value gives the beta amplitude and the argument gives the beta phase. Second, this matrix was used to fit the parameters describing the complex multivariate Gaussian distribution [65]: p(x) = β exp[ − 1/2 (x_B_ − μ_B_)^H^ R_B_^−1^ (x_B_ − μ_B_)], where x is a N_channel_ × 1 vector of complex values, x_B_ = [x; conj(x)] is the 2N_channel_ × 1 (augmented) vector of complex values, μ_B_ is a 2N_channel_ × 1 vector of complex values representing the mean of x_B_, R_B_ is the 2N_channel_ × 2N_channel_ (augmented) covariance matrix of x_B_, b = 1/(π^N^ sqrt(det(R_B_))) is a normalization term, conj(x) returns the complex conjugate of x, and the superscript H represents the conjugate transpose operation. Call this distribution, fit using all data, the baseline distribution p_BASE_(x). Third, perform another distribution fitting using LFP data from spike times only; call this the spike-triggered distribution p_ST_(x). Fourth, from a new training dataset of filtered LFP signals, extract the N_channel_ × 1 vector representing each sample point and compute the log-likelihood ratio L(x) = log[p_ST_(x)/p_BASE_(x)], generating a 1 × N_samples_ time series of log-likelihood ratio values. Call this time series L. Fifth, compute the L-to-rate mapping for this training dataset, as was described above for the amplitude-to-rate mapping. Sixth, find the best 4-parameter sigmoid fit F_S_(L | p) for the L-to-rate mapping, where p is a 4 × 1 parameter vector (see sigmoid function definition above). Seventh, given a novel test dataset of filtered LFP signals, extract the N_channel_ × 1 vector representing each sample point and compute the predicted instantaneous spike rate estimate R_EST_(x) = F_S_ [log[p_ST_(x)/p_BASE_(x)]]. Eighth, evaluate the prediction by computing the estimated-rate-to-measured-rate mapping, computed as was done to estimate the amplitude-to-rate mapping.

## Results

We first consider the mapping from beta amplitude alone to spike rate (amplitude-to-rate mapping), then from beta phase alone to spike rate (phase-to-rate mapping), before examining the joint influence of beta amplitude and phase on neuronal spiking. Both the amplitude-to-rate and phase-to-rate mappings exhibit task-dependent changes, as does the full beta-to-rate mapping, and we characterize the distribution of mapping parameters across the population. Next, we consider the dependence between spiking and the beta phase difference between left and right primary motor cortices; whereas amplitude and phase provide information about a single area, the phase difference provides macroscopic information about the relationship between areas. Finally, we consider the issue of spike dependence on meso-scale spatial patterns, and the effect that task-dependent changes have on the predictability of spiking.

### Beta amplitude-to-rate mapping

**Figure 3:**
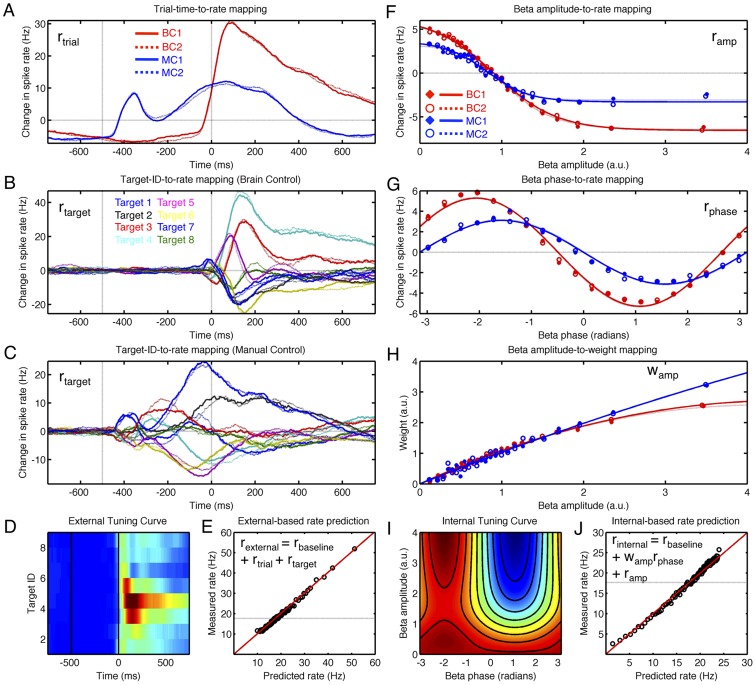
External and internal tuning curves. Tuning curves characterize neural properties by conditioning spike rates on external factors such as movement direction or internal factors such as beta amplitude or phase. A–E show the external tuning properties for one neuron (sig045a), while F–J characterize the internal tuning properties for the same cell. A) Trial-related rate changes relative to baseline, collapsed across all targets. Go-cue onset is 0 ms. Four disjoint datasets are shown (BC1 & BC2, red; MC1 & MC2, blue). B) Target-specific rate changes (relative to baseline and trial-related activity) for the 8 BC targets. Solid lines show responses for BC1, dotted lines BC2. C) As in B, for MC. D) External tuning: joint display of trial- and target-related rate changes in BC1; color indicates spike rate. E) External tuning components (r_baseline_, r_trial_, and r_target_) are learned from training data (BC1) and applied to novel test data (BC2) to predict instantaneous spike rate. F) Rate changes associated with different beta amplitudes. Beta amplitude normalized to a mean of 1. For this neuron, large beta amplitudes are associated with reduced firing and low amplitudes with increased spiking, but rate of change is task-dependent. G) As in F, conditioning spike rate on beta phase rather amplitude. H) Weight term governing the interaction between amplitude and phase (see Materials and Methods). I) Internal tuning: joint display of amplitude- and phase-related rate changes in BC1; color indicates spike rate. J) Internal tuning components (r_baseline_, r_amp_, r_phase_, and w_amp_) are learned from training data (BC1) and applied to novel test data (BC2) to predict instantaneous rate.

**Figure 4:**
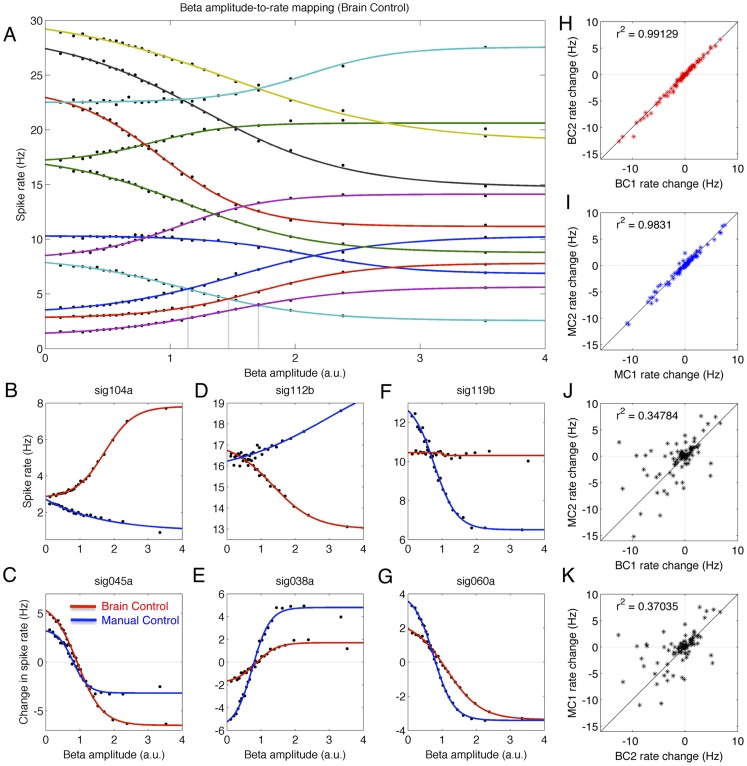
Beta amplitude-to-rate mapping. A) A diversity of amplitude-to-rate mappings hold across neurons during a given task; shown are 12 example neurons during BC. Dots indicate measured spike rates, lines show best-fit sigmoids. Increased beta amplitude associated with decreased rate in some neurons while others exhibit increased firing; vertical lines indicate cross-over points associated with change in firing-rate rank order within ensemble. B–G) Amplitude-to-rate mapping can change as function of task; six example neurons shown. H) Within-task CLC parameter stability assessed by computing amplitude-to-rate mapping for disjoint BC datasets; positive (negative) rate changes indicate that spike probability and amplitude are positively (negatively) correlated. I) As in H, for MC. J–K) Direct comparison of BC/MC datasets provides evidence for cross-task remapping; the amplitude-to-rate mapping for one task may not hold for different task. Similarity of J and K indicates reliable task-dependent remapping.

Given the strong event-related changes in beta amplitude during both MC and BC (Figure 2B,E), we first investigated the dependence of spike rates on beta amplitude alone (neglecting beta phase or beta phase differences between sites). We term this functional dependence between beta amplitude and spike rate the amplitude-to-rate mapping, consistent with the idea that a given neuron responds to both internal and external factors (Figure 3). This analysis revealed two key findings. First, within a given task such as BC, the population of simultaneously-recorded neurons exhibited a wide range of responses to changes in beta amplitude, with some neurons increasing firing, some exhibiting no change, and other decreasing their spike rate (Figures 4A,H–I; S2A–F; S10). Second, a single neuron may exhibit a task-dependent remapping of the amplitude-to-rate function – for example, a given neuron may increase firing as beta amplitude goes up during BC, but decrease firing when beta amplitude increases during MC (Figures 4B–G,J–K; S1A–F; S2G–H). In more detail below, we consider i) the diversity of within-task amplitude-to-rate mappings observed across the full neuronal ensemble, and ii) the diversity of amplitude-to-rate remappings that can occur within a single neuron when switching from one task to another.

Across both the MC and BC tasks, a statistical dependence between spike rate and beta amplitude was observed for 86.7% of the cortical motor neurons examined (p<0.01 uncorrected randomized permutation test; c.f. Table S1). That is, for a given neuron the spike density conditioned on low beta amplitude is different than the spike density conditioned on high beta amplitude. We found that the empirically-observed amplitude-to-rate mapping was well-described by a 4-parameter sigmoidal function (see Materials and Methods). Across the neuronal population, this mapping was described by a wide range of model parameters. For example, Table S1 shows that as beta amplitude increased during BC, 58.6% of all neurons exhibited a decrease in spike rate while 28.2% showed an increase in spike rate. As beta amplitude increased during MC, 35.4% (40.3%) of neurons decreased (increased) their firing rate. Importantly, while the neuronal population exhibited a wide range of parameter values during a given task, the parameters for single neurons showed high stability across different sessions of the same task (Figures 4H,I; S2C,F).

Figure 4A shows the range of amplitude-to-rate mappings during BC for twelve example neurons from monkey P. Dots show the empirically-observed spike rates conditioned on beta amplitude, computed separately for 25 non-overlapping amplitude bins. Note that adaptive binning was employed such that each bin includes the same number of sample points, resulting in non-uniform bin spacing. Lines indicate the best-fit sigmoidal functions describing the observed amplitude-to-rate mapping (see Materials and Methods). Some neurons show a decrease in spike density with increasing beta amplitude while others show an increase in spike density. Furthermore, as shown for a different set of neurons in Figure S10, removing the offsets due to baseline spike rates reveals that the rate of change of the mapping (neglecting sign) is large for some neurons (purple, gold), moderate for others (green, cyan), and small for yet others (black, red). Critically, for a given neuron the sign and slope of the amplitude-to-rate mapping is not correlated with the cell's baseline firing rate, and are different for distinct cells. Thus, a change in beta amplitude does not imply a stereotyped change in spike density that applies to all neurons uniformly; different neurons exhibit differential responses to changes in beta amplitude. That is, while averaging across all recorded cells reveals a negative correlation between ensemble spike rate and beta amplitude, investigating each cell separately reveals that some neurons exhibit a strong negative correlation with beta amplitude, others a strong positive correlation, while yet others show only a weak or negligible dependence on amplitude (c.f. Figure 4H, I).

Interestingly, while there is variation in the exact crossover point for the population of amplitude-to-rate mappings – that is, the amplitude value where the sigmoid function intersects the baseline rate – the amplitude-to-rate mappings for many neurons intersect near the mean beta amplitude (Figure S10). In all analyses presented here the mean beta amplitude has been normalized to 1. However, while most baseline-free amplitude-to-rate mappings intersect near the same beta amplitude (the mean value), adding different baseline rates can shift the crossover point for pairs of amplitude-to-rate mappings. For example, Figure 4A shows the intersection of the amplitude-to-rate mappings for neuron sig060a (cyan, bottom) with the amplitude-to-rate mappings of three other cells: sig099a (blue), sig104a (orange), and sig031a (purple). Vertical lines mark the amplitude values where these curves intersect. For low beta amplitudes, sig060a (cyan) has a higher spike rate than the other 3 neurons, while for high beta amplitudes this neuron has a lower rate. For intermediate amplitude values sig060a has a higher rate than some neurons but not others. Large differences in baseline rates result in no overlap of amplitude-to-rate functions and thus no change in the relative rank ordering of neurons in terms of spike rate.

Thus, for an ensemble of neurons with similar baseline rates, the common intersection point of the amplitude-to-rate mappings near the mean beta amplitude results in two distinct spike density regimes for the ensemble. That is, when the instantaneous beta amplitude is above its mean value, then there is an associated rank ordering of the spike rates across the population of neurons (relative to the tonic baseline rate for each neuron). For example, given the seven example neurons shown in Figure S10, a beta amplitude above the mean is associated with the rank order gold, cyan, red, blue, black, green, and purple (ranked highest-to-lowest in terms of change in spike rate relative to the baseline rate for each neuron). In contrast, when the instantaneous beta amplitude is below the mean, then this rank ordering is reversed. However, for neurons with different tonic baseline spike rates (Figure 4A), the amplitude-to-rate mappings of each pair of neurons may cross at beta amplitude values far from the mean value. That is, each pair of neurons in an ensemble may switch their spike rate rank order at any of a wide range of amplitudes, greatly expanding the set of rank-order states possible for the ensemble. Furthermore, each rank-order ensemble state – where an ensemble state is defined as an ordered list of neurons sorted in terms of spike rate – is indexed by a finite range of beta amplitudes. The vertical lines in Figure 4A show three transition points between such states; across both BC and MC conditions, the amplitude-to-rate mappings for the 12 neurons shown in Figure 4A and Figure S2 establish a set of 41 such states, each of which is associated with a finite interval of beta amplitudes.

The within-task diversity of amplitude-to-rate mappings observed across the ensemble of recorded neurons is complemented by a different type of diversity – within-neuron, cross-task diversity – that is associated with task switching. Interestingly, while the amplitude-to-rate mappings for a given neuron are relatively stable across multiple datasets as long as all recordings are acquired under the same task conditions (Figures 4H,I), switching from one task to another is associated with a reliable remapping of the amplitude-to-rate relation within a single neuron (Figures 4B–G,J,K; S1A–F, S2). That is, a given neuron may exhibit one stable amplitude-to-rate mapping during a session performed under MC, then switch to a distinct (and stable) amplitude-to-rate mapping during a second session conducted under BC, and finally return to the original amplitude-to-rate mapping when performing a third session under MC. Figures 4B–G show six example neurons that give an indication of the range of task-dependent remapping that can occur. For example, the single unit sig119b (Figure 4F) shows a strong decrease in spike density associated with increasing beta amplitude during MC (blue), but little to no change in spike density during BC (red). Unit sig045a (Figure 4C) shows a different response, with a moderate decrease in spike rate during MC but a large decrease in rate under BC. Unit sig038a (Figure 4E) exhibits a positive correlation between spike rate and beta amplitude, with a larger total change of rate under MC. Other cells exhibit the direction reversal shown by units sig104a (Figure 4B) and sig112b (Figure 4D), with rate increasing under one task and decreasing in another.

As shown by Table S2, of the 64.1% (116/181) of neurons across both monkeys that exhibited significant amplitude-to-rate mappings during both MC and BC, 27.6% showed a direction reversal when comparing the MC and BC amplitude-to-rate mappings (similar to neuron sig104a in Figure 4B). Of the cells positively correlated with beta amplitude under both BC and MC, 61.8% of cells exhibited a larger modulation depth for MC compared to BC (similar to neuron sig038a in Figure 4E), while the remaining 38.2% showed a smaller modulation depth for MC compared to BC. For the 43.1% of cells exhibiting a negative correlation between rate and amplitude for both MC and BC, 56.0% of cells exhibited a larger modulation depth for MC compared to BC, while the remaining 44.0% showed a larger modulation depth for BC compared to MC (similar to neuron sig045a in Figure 4C).

In addition to task-dependent remapping, the amplitude-to-rate mapping also exhibits changes as a function of trial-stage, as shown for three example neurons in Figure S9 (c.f., Figure S5). Importantly, task-related differences are observed during similar trial sub-stages. For example, considering data from segments of the trial where the goal was to move the cursor to the center cue, or toward one of the peripheral targets, still resulted in differences in the amplitude-to-rate mapping between MC and BC (Figure S9A–B, D–E, G–H). Periods where goal-directed activity was presumably minimized, such as the period around reward delivery, also resulted in task-related differences (Figure S9C, F, I). This suggests that task-related changes are distinct from trial-substage related changes, although further experiments will be required to fully disentangle the influence of top-down task demands from bottom-up trial-stage-related changes on the amplitude-to-rate mapping.

Importantly, both the within-task amplitude-to-rate mappings and the cross-task amplitude-to-rate remappings are stable across multiple data sets (Figure 4H–K). For example, Figures 4H–I shows the stability of these mappings when the data is divided into two disjoint datasets for each task, and the amplitude-to-rate mappings are computed separately for each dataset. That is, the correlation between parameters defining the amplitude-to-rate mappings computed from two blocks of data from the same task (BC1/BC2, Figure 4H, red; MC1/MC2, Figure 4I, blue) is higher than the parameter correlation between amplitude-to-rate mappings estimated from different tasks (BC1/MC2, Figure 4J; BC2:MC1, Figure 4K). Therefore, the distribution of amplitude-to-rate mappings observed across the neuronal ensemble is stable from one dataset to another as long as the same task is being performed (within-task stability of mappings), but exhibits a reliable and reversible shift when switching from one task to another (cross-task reliability and reversibility of remappings). This association of the beta amplitude-to-rate mapping with an ordered sequence of discrete ensemble states – and the ability to create a new sequence via task-dependent remapping of the amplitude-to-rate relations that hold within an ensemble – has intriguing implications for neuronal computation, which we explore further in the discussion.

### Beta phase-to-rate mapping

**Figure 5:**
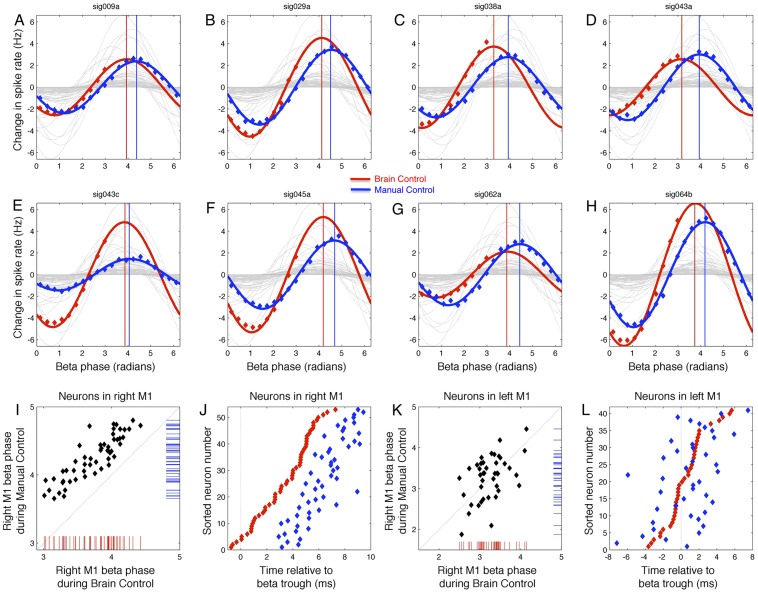
Beta phase-to-rate mapping. A–H) Eight example neurons that exhibit task-dependent remapping of beta phase-to-rate relationship; fits for all neurons shown in grey. Vertical lines indicate phase of maximal spiking for BC (red) and MC (blue). Preferred phase varies across neurons within a task, but all BC phases occur earlier than preferred MC phases. I) Preferred phase for BC vs. MC for all 53 neurons in right M1, exhibiting task-dependent shift to later phase for MC. J) Preferred beta phases map to times of peak spike probability relative to the ongoing beta rhythm; shown are peak times for all neurons in right M1, sorted relative to beta trough during BC (red). MC (blue) does not preserve the BC ensemble timing order. K) As in I, for neurons in left M1. L) As in J, for left M1.

As with beta amplitude, cortical motor neurons exhibited a spike density dependence on beta phase. During BC (MC), the spike density of 87.3% (91.2%) of neurons exhibited cosine modulation when conditioned on beta phase (p<0.01, uncorrected randomized permutation test). That is, when considering beta phase alone (neglecting beta amplitude for now, but see beta-to-rate mapping below), the change in spike rate as a function of beta phase (phase-to-rate mapping) was well-fit by a 3-parameter cosine-type function (see Materials and Methods). Figures 5A–H show eight example neurons recorded from right primary motor cortex (M1) that exhibit this phase-to-rate mapping for BC (red) and MC (blue), superimposed on the fits for all 95 simultaneously-observed neurons from monkey P (grey). For all neurons that exhibited sensitivity to beta phase, this mapping was unimodal, with no neurons exhibiting multimodal dependence on beta phase. As with the amplitude-to-rate mappings, most neurons exhibited stable within-task phase-to-rate mappings (evaluated using different datasets recorded under the same task conditions) as well as reliable and reversible cross-task phase-to-rate remappings (evaluated using datasets recorded under BC or MC; c.f. Figures S4).

For a given neuron, both the modulation depth (maximum rate minus baseline rate) and preferred beta phase (beta phase exhibiting the maximum spike rate) could change from one task to another. For example, some neurons show few cross-task changes (e.g., Figure 5A), while others primarily exhibit a change in preferred phase alone (Figure 5D), or a change in modulation depth alone (Figure 5E), or a change in both modulation depth and preferred phase (Figure 5F). Interestingly, for all 53 neurons from right M1 of monkey P, the preferred beta phase during BC was earlier than the preferred phase under MC, as shown in Figure 5I. This systematic shift in preferred beta phase was uncorrelated to the tonic baseline firing rates of neurons in either task (Figure S3D), and was also uncorrelated with the change in baseline rates from one task to another. In other words, this systematic shift in preferred phase is not due to a simple rate-to-phase conversion of the type shown in *in vitro* studies [66]. Furthermore, this preferred beta phase was unrelated to the strength of motor direction tuning or preferred movement direction (Figure S3H, L).

Given the peak in the LFP power spectrum at a center frequency of 28 Hz, the average beta cycle occurs over ∼36 ms. Therefore, we can convert a set of preferred phases into a set of most-probable spike times relative to a fixed point in the cycle of the ongoing beta rhythm. That is, if a neuron is going to spike only once in a given beta cycle, it is most likely to do so at its preferred beta phase, which corresponds to a fixed temporal lag relative to the peak of the beta waveform. The ordered set of these lags across the population imposes a probabilistic rank-ordering of spike times across the ensemble that spans ∼10 ms (sorted red dots in Figure 5J and 5L). This is not a strong deterministic ordering of spike times but is rather a probabilistic or stochastic effect. Nonetheless, the knowledge of the preferred beta phase for a pair of cells can be informative about their relative spike timing. As a concrete example, consider neurons sig038a (Figure 5C) and sig045a (Figure 5F). The phase-to-rate mapping for cell sig038a peaks earlier within the beta cycle than does the phase-to-rate mapping for sig045a (3.42 radians vs. 4.22 radians, respectively). Therefore, given a particular cycle where each cell spikes exactly once during the cycle, we would expect sig038a to spike earlier than sig045a. In fact, considering only the cycles where each cell fires exactly once, sig038a fires before sig045a 61.0% (40684/66678) of the time. Interestingly, however, due to the ensemble-wide, task-dependent shifts in the preferred beta phases, several pairs of neurons exchange their most probable firing order when switching from one task to another. For example, the unit sig064b (Figure 5H) is most likely to fire before unit sig043c (Figure 5E) during BC (red), but the order switches during MC (blue).

**Figure 6:**
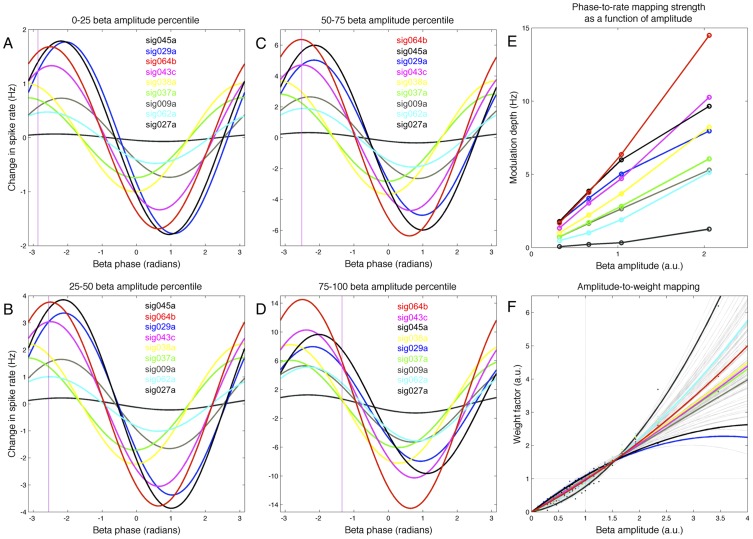
Modulation of phase-to-rate mapping by beta amplitude. Beta phase has a stronger impact on spike rate when beta amplitude is large, but gain modulation is not uniform across neurons. A–D show the phase-to-rate mappings for 9 example neurons, where instantaneous phases and spike times were pre-sorted into one of four bins based on beta amplitude (see Materials and Methods). Phase-to-rate modulation depth (difference between maximum and baseline rates) is largest for bin with largest amplitudes (c.f. scale of y-axis of A–D), but some modulation depth increases faster for some neurons than others; sig045a (black) has highest rate in smallest amplitude bin but ranks third in the largest amplitude bin, while sig043c (cyan) moves from rank 4 to 2. Differential changes can shift the beta phase where two neurons exchange spike rate rank order, even if the preferred phases do not change (note shift in phase of crossover of sig043c (cyan) with sig029a (blue), indicated by vertical lines. E) Phase-to-rate modulation depth as a function of mean beta amplitude. F) Amplitude modulation of phase-to-rate mapping can be expressed as a product of two terms, one of which is a quadratic weight factor (see Materials and Methods).

Finally, the phase-to-rate mapping is strongly affected by the magnitude of beta amplitude (Figure 6). For example, Figures 5A–D show the phase-to-rate mappings for 9 example cells, computed separately for data falling into 4 beta amplitude bins (corresponding to 0–25, 25–50, 50–75, and 75–100^th^ amplitude percentiles). The relative sizes of the phase-to-rate mappings shown in Figures 5A–D are largely similar across the four amplitude bins, with all cells exhibiting an increase in phase-to-rate modulation depth (note vertical scale in Figure 6A–D). This increase phase-to-rate modulation depth as a function of beta amplitude is shown explicitly in Figure 6E. However, all cells do not change their modulation depth at the same rate – as beta amplitude increases, some cells increase their phase-to-rate modulation depth at a faster rate than others. Thus, while sig045a (black) starts out with the largest firing rate at low beta amplitudes, at high beta amplitudes it has fallen to rank 3 among the 9 neurons shown. Similarly, sig029a (blue) falls from rank 2 to rank 5, while sig043c (purple) moves up from rank 4 to rank 2. In addition to these differential changes in total phase-to-rate modulation depth as function of beta amplitude, there can also be shifts in the phase value associated with the crossover point of the phase-to-rate mappings for a pair of neurons. This can be the case even if each neuron maintains the same preferred beta angle. For example, the phase-to-rate mappings for sig043c (purple) and sig029a (blue) cross near −3 radians at low beta amplitudes (vertical line), but shifts to approximately −1.3 radians for high beta amplitudes, even though the preferred beta phase for each cell remains the same. The cosine-shaped phase-to-rate mapping is a nonlinear function of phase, and combined with differential changes in vertical scale as a function of amplitude, these exchanges of spike-rate rank order as a function of beta phase can occur even when each cell has no change in its preferred beta phase angle.

While the (within-task) phase-to-rate preferred beta angle is largely stable for most neurons across the full range of beta amplitudes (Figure S4), the change in phase-to-rate modulation depth can be described by a quadratic function of beta amplitude (Figure 6F). We call this term describing the gain control of the phase-to-rate modulation depth the weight factor or the amplitude-to-weight mapping. This weight factor is sublinear function of amplitude for some neurons (sig029a, blue), near linear for others (sig038a, yellow), and supralinear for yet others (sig027a, black). Despite this variability, across all neurons larger beta amplitudes are associated with larger modulation depths. That is, beta amplitude appears to act as a gain control for beta phase, such that beta phase is more predictive of spike timing when beta power is high than when beta power is low. However, just as the heterogeneity of tonic baseline rates and sigmoidal amplitude-to-rate functions interact to establish an ordered set of ensemble rank-order states that are indexed by beta amplitude, ensemble heterogeneity in phase-to-rate modulation depth and the quadratic weight factor can interact to establish an ordered set of rank-order states indexed by beta phase. That is, for a given beta amplitude, a set of overlapping phase-to-rate mappings have crossover points that occur at particular beta phases (vertical line in Figure 6A). As beta amplitude changes, however, these crossover points can shift to new phases (vertical line in Figure 6D). Furthermore, different tonic baseline rates can force some crossover points to disappear or introduce new crossings. Thus, both the amplitude-to-rate and phase-to-rate mappings, considered across an ensemble of neurons, can be associated with ordered sets of neurons, where cells are sorted according to instantaneous spike rate.

Nonetheless, one consequence of this interaction between amplitude and the phase-to-rate modulation depth is that the spike timing preference relative to beta phase becomes stronger for higher beta power. For example, we saw above that neuron sig038a fires before neuron sig045a 61.0% percent of the time when looking at all beta cycles where each cell fires once. Sorting these individual cycles according to beta amplitude, however, reveals that sig038a spikes before sig045a only 56.2% of the time for cycles in the lowest decile beta amplitudes, compared to 72.0% of the time for the cycles in the highest decile of beta amplitudes. Similar effects are seen across the population, and thus variations in beta amplitude influence the probability of observing arbitrarily spike timing sequences within an ensemble.

**Figure 7:**
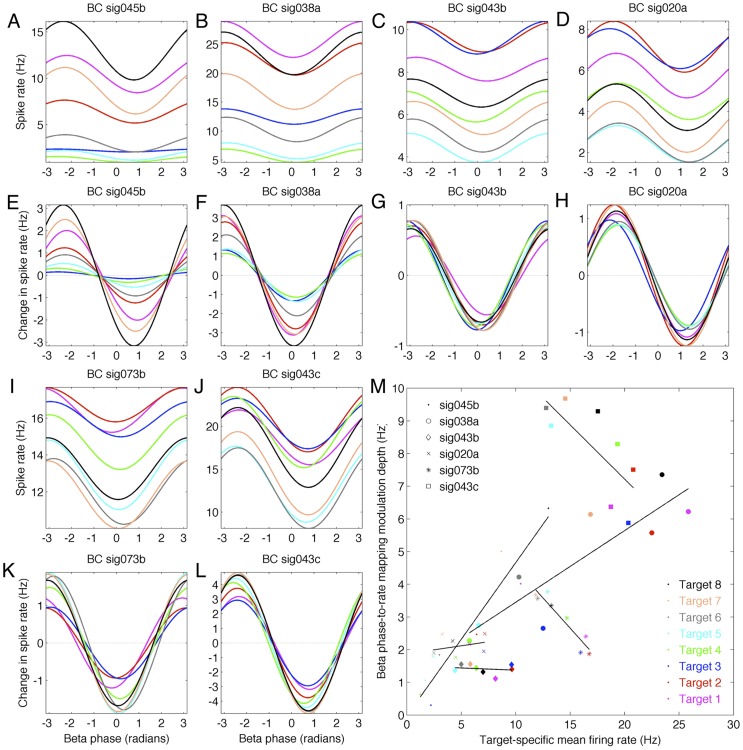
Target-specific modulation of phase-to-rate modulation depth. Sorting trials based on the intended BC target prior to computing the phase-to-rate mapping reveals differences in baseline firing (due to direction tuning) as well as changes in the phase-to-rate modulation depth. A–L show 6 example neurons where the phase-to-rate modulation depth is positively correlated with the target-specific shift in baseline spike rate. Colors indicate phase-to-rate mappings computed from trials moving toward different targets. Shown are phase-to-rate mappings with target-specific baseline shifts included (A–D, I, J) or removed (E–H, K, L). For example, sig045b (A, E) fires the most for Target 8 (black) and the least for Target 4 (green), and also exhibits the largest phase-to-rate modulation depth for Target 8 and the least for Target 4 – that is, target-specific spike rates and phase-to-rate modulation depth are positively correlated (c.f. sig038a in B, F). In contrast, the phase-to-rate modulation depth is largely independent of target direction for sig043b and sig020a, despite the large target-specific shift in baseline spike rates. Finally, sig073b and sig043c provide examples of negative correlation between target-specific shifts in baseline spike rates and target-specific changes in phase-to-rate modulation depth. Correlations for these 6 examples are shown in M.

As with the amplitude-to-rate mapping, the phase-to-rate mapping exhibits both task- and trial-stage-related changes (Figures S6, S7). As shown by Figure 3, neurons respond to both internal and external factors, including time-in-trial and target direction. One possibility is that changes in the amplitude- and phase-to-rate mappings may arise from the interaction of multiple external and internal factors each of which influences the overall spike rate. For example, Figures 3B–C show that the target-specific spike rate for neurons can span a wide range of values from the most preferred to least preferred targets. Similarly, Figure 7 shows the target-specific phase-to-rate mappings for 6 example neurons during BC. Each neuron exhibits changes in the baseline rate that is a function of target direction or target ID. For example, Figure 7A shows that sig045b has the highest baseline rate for Target 8 (black) and the lowest baseline rate for Target 4 (green). However, removing this target-specific baseline offset, as done for the same cell in Figure 7E, shows that the phase-to-rate modulation depth exhibits target-specific changes as well – in fact, the target-specific baseline rates of sig045b are positively correlated with the target-specific changes in the phase-to-rate modulation depth (Figure 7M, dots). Similarly, sig038a exhibits target-specific variation in both the baseline rates (Figure 7B) and the phase-to-rate modulation depth (Figure 7F), also with a positive correlated between them (Figures 7M, circles). However, sig043b and sig020a exhibit little target-specific variation in the phase-to-rate modulation depth (Figures 7G, H) despite strong target-specific variation of their baseline firing (Figures 7C,D). In fact, the slope of the regression line between target-specific baseline rates and modulation depths is close to zero (Figure 7M, diamonds and crosses). On the other hand, cells such as sig073b and sig043c exhibit a negative correlation (Figure 7M, asterisks and squares). Thus, while the amplitude-to-rate mapping may exhibit target-specific changes, the functional nature of this relationship remains unclear.

Because the spike density of a given neuron depends on the interaction between beta amplitude and phase, the most complete picture of the dependence between spike rates and the beta rhythm is given by the full beta-to-rate mapping (where the term ‘beta’ here implies both beta amplitude and beta phase). That is, the estimated spike rate R_BETA_(a,θ) is a sum of two terms: an amplitude-only rate R_AMP_(a) given by the amplitude-to-rate mapping, and another term that is the product of the phase-only rate R_PHASE_(θ) and a weight factor that is a function of amplitude alone, w_AMP_(a). Specifically,
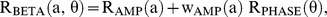
where R_AMP_(a) is a sigmoidal function of amplitude, w_AMP_(a) is a quadratic function of amplitude, and R_PHASE_(θ) is a weighted cosine function of phase (see Materials and Methods). Alternatively,
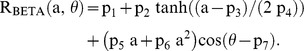
appears to provide a good fit to data across the ensemble, given sufficient data. The primary result of this study is the finding that parameters describing this beta-to-rate mapping can change reversibly from one set of stable values to a different set of stable values when switching from one task to another.

**Figure 8:**
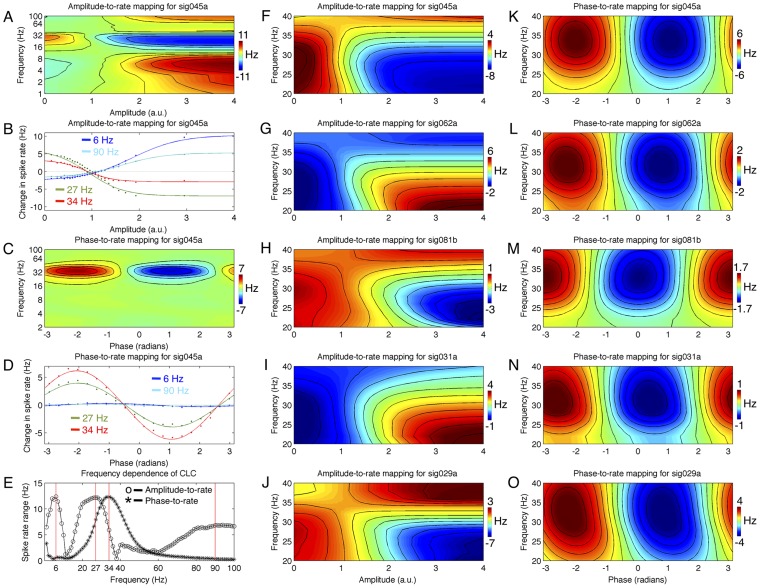
Frequency dependence of amplitude- and phase-to-rate mappings. A) Amplitude-to-rate mapping for one neuron (sig045a), computed for a range of center frequencies (1–100 Hz). Vertical axis gives filter center frequency, horizontal axis gives amplitude at that center frequency (normalized to a mean of 1 for all frequencies); color gives spike rate change relative to baseline. This neuron exhibits different responses for different frequencies; positive correlation of rate with theta and high gamma bands, but negative correlation with beta and low gamma. B) Same data as A, showing only four frequency bands at 6, 27, 34, and 90 Hz. Dots indicate measured rates, lines are best fit sigmoids. C) As in A, for the phase-to-rate mapping. Strongest response for this neuron is seen at 34 Hz for this neuron. D) As in B, for the phase-to-rate mapping. E) Range of rate change for sig045a as function of center frequency. Peaks of the amplitude- and phase-to-rate ranges are offset, with the amplitude-to-rate mapping strongest ∼27 Hz while the phase-to-rate mapping is strongest ∼34 Hz. F) As in A, for a finer frequency resolution from 20–40 Hz. G–J) As in F, for neurons sig062a, sig081b, sig031a, and sig029a. K) As in C, from 20 to 40 Hz for neuron sig045a. L–O) As in K, for neurons sig062a, sig081b, sig031a, and sig029a.

However, while the beta rhythm exhibits strong event-related changes in power (c.f., Figure 2), it is possible that different frequency bands may prove even more predictive of cell spiking. An example of this is provided for neuron sig045a in Figure 8A, where the amplitude-to-rate mappings for a wide range of frequencies from 1–300 Hz are shown. Figure 8B shows the amplitude-to-rate mappings for four different center frequencies. For this cell, a center frequency of 6 Hz is as informative as is 27 Hz. However, as shown by Figures 8C–D, for this cell the informative phase-to-rate mappings are restricted to a narrow range of frequencies centered around 34 Hz. Figure 8E shows the range of spike rate variation for this cell as a function of center frequency for both the amplitude- and phase-to rate mappings. Interestingly, the amplitude- and phase-to-rate mappings appear to peak at different but possibly overlapping bands. The center frequency used for this study, 28 Hz, intersects both profiles near their peak, and thus provides information from both types of mapping. Figures 8F–O provide amplitude- and phase-to-rate mappings for 5 example cells over a range of center frequencies from 20 to 40 Hz, showing that the best phase-to-rate center frequency is often higher than the best amplitude-to-rate center frequency – a result that holds across the ensemble. The importance of optimal phase-to-rate center frequencies ∼30–38 Hz, however, is difficult to reconcile with the lack of strong event-related power changes (Figure 2B, E) or event-related phase-resetting (not shown) in this band. Whether or not the amplitude- and phase-to-rate mappings arise from distinct bands will require further experimental inquiry targeting this question.

### Beta phase-difference-to-rate mapping

So far we have only considered the relation between (micro-scale) spiking of single neurons to (meso-scale) beta LFP activity averaged locally over several millimeters. That is, for a neuron in left M1, we examine the relation of its spike rate to the average beta activity recorded in left M1, while for neurons recorded in right M1 we examine the average field potential activity from right M1. The 8 × 8 electrode arrays used here cover 3.5 × 3.75 mm^2^, such that the LFP signals recorded on opposite sides of the array are generated by distinct cell populations, and the spatial average of all 64 LFP signals from one array is a meso-scale signal similar in scale to the activity recorded from one electrocorticography (ECoG) electrode as employed for human neurosurgery. The results above show a clear dependence between micro- and meso-scale phenomena. However, the relation between micro-scale spiking activity and fully macro-scale phenomena – such as phase coupling between the left and right motor cortices – remains unclear. Are some neurons sensitive to the phase difference between left and right M1, above and beyond the influence that can be attributed to locally-generated field potential activity?

**Figure 9:**
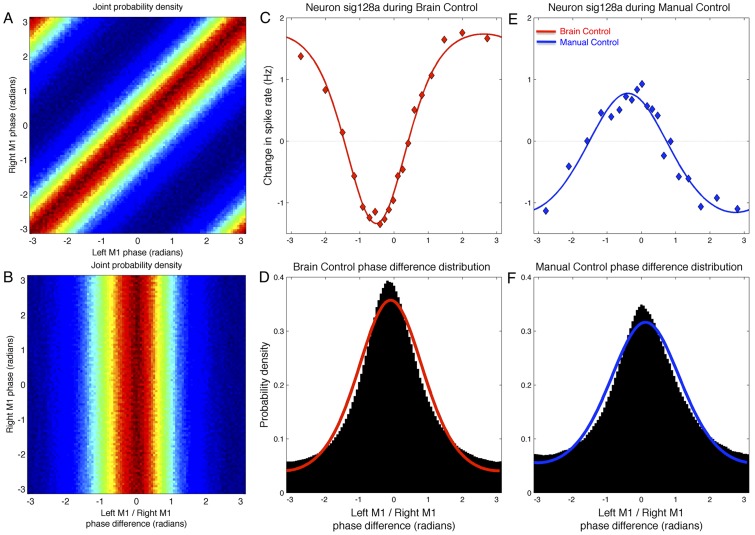
Phase-difference-to-rate mapping. A) Joint probability density function of instantaneous phases of left and right primary motor cortex (M1). B) Same data as A, after change of variables to isolate the phase difference between left and right M1. Inter-hemispheric phase differences are statistically independent from local M1 phase. C) Example neuron sensitive to beta phase difference between left and right M1. During BC, lowest rate occurs near the most probable phase difference (peak of distribution in D), but the neuron increases spiking when the inter-hemispheric phase difference shifts to less probable values. Dots indicate measured rate, red line is best-fit von Mises type function (see Materials and Methods). D) Distribution of inter-hemispheric phase differences (left M1 phase minus right M1 phase) during BC; empirical histogram estimate (black) and best-fit von Mises distribution (red). E) Same neuron as C, during MC. Despite only a small shift in the phase-difference distribution, the phase-difference-to-rate mapping has flipped for this cell. F) As in D, for MC.

To address this question, we examined the relationship between (micro-scale) single-unit spiking and the (macro-scale) relative phase difference between the beta activity occurring in the left and right primary motor cortices. While this quantity neglects the beta amplitude in each area, it has the advantage of being statistically independent of the (absolute) beta phase local to the neuron. That is, knowing the instantaneous beta phase local to the neuron alone tells us nothing about the instantaneous beta phase in the other hemisphere; however, if we know the local (absolute) beta phase as well as the relative phase difference between the hemispheres, then we can calculate the distal (absolute) beta phase in the other hemisphere (Figure 9A–B). Thus, if the mapping from the phase difference between left and right M1 to the spike rate of a neuron – the phase-difference-to-rate mapping – is non-uniform, then we can infer that distal phase information is informative about the spike rate of a neuron above and beyond the information gained by knowledge of the locally-generated beta phase (that is, beta activity generated near the soma).

Figure 9 shows an example of one such neuron (sig128a in left M1) that exhibits a non-uniform phase-difference-to-rate mapping, as well as task-dependent changes in this mapping. Figure 9D shows the distribution of relative phase differences between the left and right hemispheres during BC, while Figure 9F shows the distribution of beta phase differences under MC. Unlike the distribution of (single-channel) absolute phases, which are almost always uniform, the distribution of relative phase differences is often peaked, indicating that the two signals are coupled, either directly or via connections to other, additional areas. While there is a slight shift in the peak of the distribution when moving from one task to the other, both distributions are centered around 0 radians, indicating a tendency to zero-lag phase coupling in both MC and BC sessions.

In contrast to the relative stability of the phase-difference distribution across different tasks, the phase-difference-to-rate mappings in Figure 9C–D show that: 1) sig128a is sensitive to the relative beta phase difference between the hemispheres, above and beyond the effects of the local beta phase, and 2) the phase-difference-to-rate mapping reverses during MC. That is, during BC the cell spikes the least when the instantaneous phase difference is near its most probable value (the distribution peak in Figure 9D), and increases its spike rate when the phase difference moves away from the peak of the distribution. In contrast, during MC the same cell spikes most near the peak of the phase-difference distribution and decreases firing when the phase difference moves to less probable values.

Over the population of recorded neurons, 68.4% exhibited significant variation in their spike rates as a function of the macro-scale, inter-hemispheric beta phase differences during BC. In order to facilitate comparisons among all recorded neurons, we computed the phase difference between left and right M1 using the average signal from all LFPs in each 8 × 8 electrode array (one array per area) – that is, we generated one time series of instantaneous phase differences against which we can examine the activity of all neurons. Interestingly, the phase-difference-to-rate mapping is often stronger when the LFP signal from an electrode proximal to the neuron is used (data not shown). However, using different pairs of LFPs for each neuron makes systematic comparisons across neurons more difficult. An alternative approach to multi-scale coupling – from macro- to meso- to micro-scale – is suggested by Figure S8, which shows a dependence between the inter-hemispheric beta phase difference between left and right M1, on the one hand, and the mean beta amplitude in each area, on the other (Figure S8B). Furthermore, Figure S8D shows that the correlation between beta amplitudes recorded in left and right M1 is dependent on the phase difference between them; that is, for one value of the inter-hemispheric phase difference, the amplitude correlation is greater than 0.5, but for other values it is near 0.1 (Figure S8D). Thus, it is possible that inter-hemispheric phase differences may influence individual cells using local beta amplitude as an intermediate variable. Nonetheless, these results suggest that single cells may receive information about activity in distal locations – either directly through monosynaptic connections, indirectly through a chain of intermediate variables, or both. Furthermore, spatially averaging field potential activity over several millimeters – as is done here in order to generate a signal comparable to that recorded with the macroelectrodes employed in human electrocorticography – may result in a loss of useful information. That is, neurons may be sensitive to meso-scale spatial patterns in addition to the average activity in a cortical area, a possibility we explore below.

### Beta spatial-pattern-to-rate mapping and task-dependent changes in neuronal predictions

**Figure 10:**
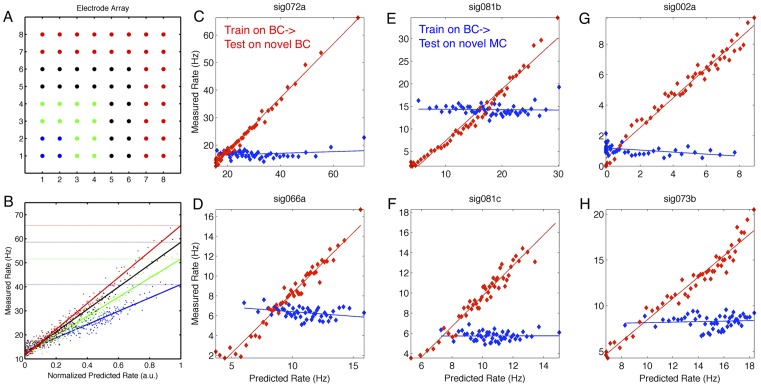
Impact of task-dependent changes predictability of spiking. A) Schematic of 8×8 microelectrode array implanted in left primary motor cortex (M1). Interelectrode separation is 500 microns on average. Color indicates groups with different numbers of electrodes, including 4 (blue), 16 (blue and green), 36 (blue, green, black), and 64 (blue, green, black, and red). B) Including more channels in a multivariate model improves prediction performance – the spike rate range increases as one considers groups of 4 (blue), 16 (green), 36 (black), or 64 (red) channels. Improvement of prediction performance suggests that distal electrodes contribute information independent of information from proximal electrodes. The predicted rate (x-axis) is shown in normalized units in order to emphasize the increase in range of the measured rate. C–H) Examples of 6 neurons where within-task predictability (red; train on BC data, test on novel BC data) is higher than cross-task predictability (blue; train on BC data, test on novel MC data). Dots indicate measured rates, lines give best linear fit. Within-task predictions are accurate for neurons across both BC and MC, implying that low cross-task predictive performance is due to task-dependent remapping rather than a lack of cross-level coupling in one of the tasks.

The analyses above employ a strictly univariate approach – the univariate (meso-scale) signal representing the mean activity in M1 is generated by spatially averaging the individual LFPs from an 8 × 8 microelectrode array (3.5 mm × 3.5 mm; c.f. Figure 10A), or the univariate (macro-scale) signal of interhemispheric phase differences is extracted. However, this approach ignores any spatial patterns that may occur on the 8 × 8 microelectrode arrays, as well as any neuron-specific preference for different spatial patterns. Furthermore, issues related to direct coupling versus indirect coupling through intermediate variables are difficult to resolve with univariate methods. Thus, investigating spatial patterns requires the use of a multivariate approach. Here we employ a method similar to that used in [41], but using complex multivariate Gaussian distributions in order to include both the amplitude and phase of multiple LFP signals. This approach uses fewer parameters per channel to characterize the relation between one LFP and the spike rate, but critically it captures the full pattern of covariances between channels and thus provides insight into the influence of meso-scale spatial patterns on the spiking of single cells.

For example, Figure 10A shows a schematic of the 8 × 8 electrode array implanted in right M1. Spikes from a single neuron are recorded on the bottom-leftmost electrode (coordinates [1,1]). Groups of electrodes more and more distal to the electrode used to record neuronal spiking are indicated in color – that is, we consider progressively larger groups of electrodes from 4 electrodes (blue), to 16 (blue and green), to 36 (blue, green, black), to 64 electrodes (all colors). Phase coupling between electrodes is a function of inter-electrode distance; with distance d in mm, the von Mises concentration parameter κ between two 28-Hz filtered signals is given by κ = 2.67 - 0.4435*d. Figure 10B shows the measured and predicted spike rates using these different-sized groups, from 4 electrodes (blue), to 16 (green), to 36 (black), to 64 (red). Importantly, the range of the measured spike rate increases as more and more channels are included. That is, as more distal electrodes are included in the predictive model and it is applied to novel data, a better prediction is generated, both in terms of the range of the measured rate as well as the coefficient of variation (r^2^). This is despite the fact that fewer parameters are used per electrode to model the cross-level coupling than for the amplitude- and phase-to-rate mappings considered above. The fact that including spatial information (in the form of more distal electrodes) improves the spike-rate predictions for individual neurons suggests that cells may be sensitive to distinct spatiotemporal patterns of population or local network activity.

Because of the stability of the within-task mapping from beta activity to single unit spiking, when given training and (novel) test data collected under the same task conditions, the spike density of individual cells can be predicted well for a large subset of neurons (c.f. [41]). Most relevant for the current study, however, is the finding that cross-task training and testing is much less effective than within-task training and testing (on novel data recorded under the same task conditions). Figures 10C–H show data from six example neurons where within-task predictions are good (red) but the cross-task predictions are poor (blue). Importantly, this loss of predictability is not due to a lack of cross-level coupling (CLC) between neurons and distributed LFP signals in one task, since CLC holds in both MC and BC tasks. Rather, while CLC occurs in both tasks, there has been a shift in the mapping to the spike rate of an individual cell from the meso-scale, spatially-averaged beta phase and amplitude, the preferred meso-scale spatial patterns, and macro-scale inter-hemispheric phase differences. That is, there is still coupling across spatial and temporal scales, but the target patterns that a particular cell is sensitive to shift when moving from one task to another. Thus, for a given neuron the cross-level coupling model obtained via training under one type of task (e.g., MC) may in fact hold little or no predictive value for the coupling observed in a different task (such as BC).

## Discussion

Above we showed that the spiking activity of neurons is coupled to multiple aspects of the motor beta rhythm during two different tasks (MC and BC), and that the form of this beta-to-rate mapping changes in a reversible, task-dependent way. For example, as beta power increases, a given neuron may increase spiking during MC but decrease spiking during BC, exhibit a reversible shift in the preferred phase of firing, or remap its sensitivity to relative phase differences between areas. This dependence on beta amplitude was well-fit by a sigmodial function (Figure 4), while the dependence of spiking on beta phase followed a cosine function (Figure 5), weighted by beta amplitude (Figure 6). These results expand on prior findings showing cross-level coupling (CLC) between spiking and LFP phase in multivariate signals [41], here showing an additional, independent coupling to beta amplitude. Critically, this work shows that cells can exhibit task-dependent changes in this coupling. Importantly, the parameters describing this beta-to-rate mapping are stable across multiple datasets of the same task (within-task stability) but exhibit reliable changes when moving from one task to another (cross-task diversity). Furthermore, we showed that the ensemble diversity of amplitude-to-rate and phase-to-rate mappings describes a set of discrete ensemble states, where each state is defined by the rank order of instantaneous spike rates. What are the implications of these empirical findings for different hypotheses about the oscillatory control of distributed networks, especially regarding local computation in a given area and long-range communication between areas?

First, there is the question of how the observed beta-to-rate mappings arise – presumably the spike activity of a subset of presynaptic cells is the origin of the amplitude- and phase-to-rate mappings for a given neuron. Rather than speculate on these origins, here we take it as given that the beta-to-rate mapping exists and instead ask what computations are now possible that are not possible or difficult if CLC is absent. We focus on two potential mechanisms that operate over different timescales: first, we consider the impact of CLC on rate-based winner-take-all (WTA) competition mediated by recurrent synaptic inhibition. Operating over a timescale of hundreds of milliseconds, modulation of WTA dynamics via the amplitude-to-rate mapping provides one link from cross-level coupling to functional neural computation. Second, operating over a timescale of tens of milliseconds, the phase-to-rate mapping biases ensemble spike timing such that some spike timing patterns are more likely than others. Through this route, cross level coupling may modulate robust temporal coding mechanisms such as synfire chain propagation.

**Figure 11:**
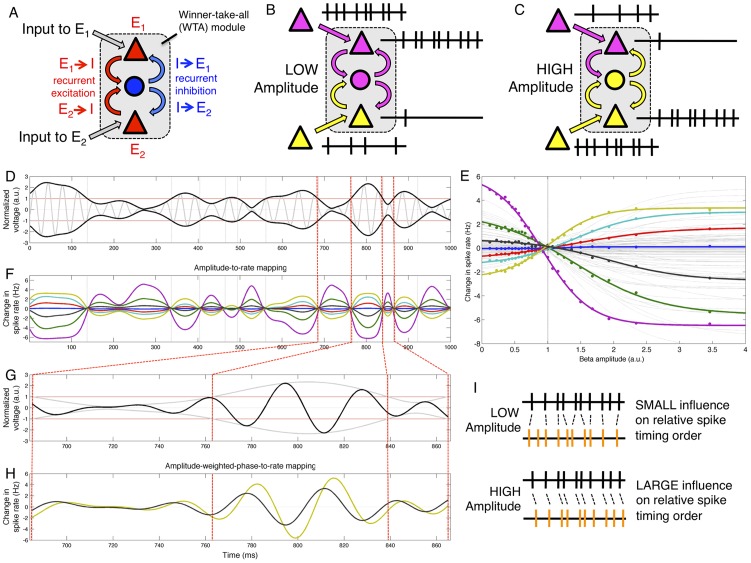
Neuro-computational consequences of amplitude- and phase-to-rate mappings. For a given neuron, the amplitude- and phase-to-rate mappings are produced by the combined synaptic input to that cell. But since information about the population rhythm is broadly accessible, neurons may use this information to dynamically organize relative activity within a functional ensemble. This activity includes winner-take-all interactions arising from recurrent local connectivity and relative spike timing among ordered sets of cells. A) Two excitatory cells (E_1_ and E_2_, red) that connect to a common inhibitory cell (I, blue) – and which in turn provides inhibitory synaptic connections to E_1_ and E_2_ to form re-entrant or recurrent excitatory-inhibitory loops – can act as a simple winner-take-all (WTA) module. That is, given different levels of input to E_1_ and E_2_, then either E_1_ or E_2_ (but not both) will produce tonic spike output. B–C) If two cells with different amplitude-to-rate mappings provide input to such a WTA module, then the WTA module will provide different output at low and high beta amplitudes. For example, given the purple (sig045a) and gold (sig062a) amplitude to rate mappings shown in Figure S10, then WTA cell E_1_ generates spike output only at low amplitudes while E_2_ spikes at high amplitudes; E_1_ and E_2_ switch roles at the beta amplitude where the amplitude-to-rate sigmoids intersect. Critically, task-dependent remapping implies that this intersection point can shift to different values for each pair of input neurons. D) One second example trace of filtered LFP activity during BC showing beta amplitude (black) and phase (grey) variation over time. E) Amplitude-to-rate mappings for seven example neurons: sig015a (blue), sig029a (green), sig029b (red), sig031a (cyan), sig045a (purple), sig062a (gold), and sig081b (black). Baseline rate has been removed to emphasize rate changes associated with amplitude variation. F) Changes in spike rates (relative to baseline) over one second induced by the amplitude-to-rate mappings (color as in E). Colors are as in Figure 4A. Note the two alternating periods of rank-ordered regimes. G) Close up of 180 ms of beta activity, showing amplitude (grey) and phase (black) variation. H) Rate changes induced by the amplitude-weighted phase-to-rate mapping for sig081a (black) and sig062a (gold). I) Periods of high beta amplitude are associated with a bias towards a relative spike timing order, while periods of low beta amplitude are not. Task-dependent remapping of preferred phases can switch this order. Task-dependent changes in the relative spike timing order of an ensemble – via the independent phase-to-rate remapping of each cell – provides a potential mechanism linking the global or top-down input changes associated with task switching to local features such as cell assembly activation or synfire chain propagation (thus influencing local cortical computation) as well as spike-timing dependent plasticity (thus influencing learning).

When evaluating different neurocomputational mechanisms, it is important to keep the anatomical facts clearly in mind in order to rule out mathematically elegant but biophysically implausible options. In this regard, the recurrent excitatory/inhibitory loops of local cortical circuits appear to provide an ideal platform for winner-take-all (WTA) dynamics [67]. Figure 11A presents a simplified schematic of a WTA module, where multiple input paths are converted into the activation of one output path via competitive di-synaptic inhibition. In this module, two excitatory cells, E_1_ and E_2_ (red triangles), are reciprocally connected to an inhibitory cell (blue circle) that receives input from both E-cells. Both E-cells also receive independent excitatory input from outside the module. None of the cells inside the WTA module need have amplitude-to-rate mappings or any beta sensitivity whatsoever. Next, assume two cells outside the WTA module provide the external excitatory input, and that both of these cells have amplitude-to-rate mappings that intersect. For example, consider the purple and gold cells in Figures 11B–C, which have amplitude-to-rate mappings as shown in Figure S10. For simplicity, assume these external cells providing WTA input are driven solely by their amplitude-to-rate mappings. Then for low beta amplitudes, the WTA cell E_1_ becomes active (Figure 11B), whereas high beta amplitudes cause E_2_ to become active (Figure 11C). In fact, the switch between E1 and E2 occurs at the beta amplitude value corresponding to the intersection of the amplitude-to-rate mappings for the purple and gold input cells. That is, the relative spike rate rank order of the cells providing input to the WTA module is transformed into tonic spiking along one of two possible output paths. Since the evidence presented here shows that within-task amplitude-to-rate mappings are stable, this binary output switch is tuned to a particular value of beta amplitude that is fixed for the duration of the task. Whenever beta amplitude sweeps through this value, this WTA switch changes state. By adding additional cells with amplitude-to-rate mappings that cross at other amplitude values, we can establish a linear, task-dependent sequence of binary WTA switches, each of which is tuned to or indexed by a different value of beta amplitude. Thus, each value of beta amplitude is associated with a binary vector that encodes the ensemble state.

Why would this be useful? First, recall the 12 cells shown in Figure 4A. On the one hand, there are 12! = 479001600 possible rank-ordered states for this set of neurons, corresponding to the number of permutations. The ability to generate sequences from such a large set of states would clearly prove computationally useful. However, it is unclear what biological mechanisms are available to quickly identify and activate an arbitrary state selected from the set of all possible ensemble states. On the other hand, if the 12 neurons have fixed baseline rates and flat amplitude-to-rate mappings, then state activation is not a problem since only one state is active at all times. Again, this case is not very computationally useful. In contrast to these extreme cases, an ensemble of neurons with a diversity of amplitude-to-rate mappings (as shown in Figure 4A) has both a variety of possible states (defined by the amplitude-to-rate crossings), as well as a method for indexing each state (every beta amplitude value corresponds to one particular ensemble state). More importantly, task-dependent remapping of the amplitude-to-rate functions provide the means to select a different set of ensemble states – where again each state is indexed by beta amplitude. That is, during one task such as BC, the continuous variation in beta amplitude maps to a discrete sequence of ensemble states (16 states for the 12 neurons shown in Figures 4A and S2A), while switching to another task such as MC maps the amplitude to a different sequence of ensemble states (24 states for the same set of neurons). In this view, across all tasks the continuous amplitude signal serves as an index function that establishes activation and transition probabilities for ensemble states. However, one task may require a different set of ensemble states than another – thus explaining the task-dependent remapping, as cross-level coupling parameters are tuned to evoke a desired set of ensemble states. In the example above, none of the 16 BC states or 24 MC states are shared across tasks (Figure S2I). Task-dependent remapping thus balances the need for a diversity of ensemble states with the requirement of a simple mechanism for sequential state activation. Therefore, combining WTA dynamics with beta-to-rate mapping and remapping seems to provide a physiologically plausible mechanism for the dynamic linking of distinct sequences of ensemble states to a common, readily-accessible signal representing the overall level of population activity – namely, the beta rhythm.

These ideas are consistent with the hypothesis that the functional role of the beta rhythm is to maintain the current computational state in a local network, protecting the local population against irrelevant or contradictory input [16]. That is, beta power remains high if no change in the local network state is needed, or if unwanted changes to local network state must be actively extinguished. Similarly, beta power drops when the local network state must change. The arrival of important but unexpected input may increase or decrease beta power, depending on context and task demands. In this view, beta is an active coordinating rhythm that helps to maintain or release selected patterns of ensemble activity. It is intriguing to speculate that task switching requires remapping coupling parameters in order to evoke a pre-learned sequence of WTA states, while learning involves optimization over the space of WTA sequences in a search for those sequences that prove most task effective. One prediction of this hypothesis is that ensemble amplitude-to-rate mappings will exhibit much more variability during learning than either before or after. The combination of WTA dynamics together with heterogeneous amplitude-to-rate mappings across an ensemble provides a specific and testable mechanism through which the beta rhythm could accomplish this goal of dynamic coordination.

Independent of possible functional roles played by the amplitude-to-rate mapping, phase-to-rate mappings may shift the relative probabilities of precisely-timed spike sequences. Simulation studies show that polychronous groups – sets of cells where activity propagates due to precise spike timing relations – can serve as the building blocks for cognitive operations such as working memory [68], and exhibit activity-dependent growth and decay useful for learning and pattern recognition [69]. Empirically, Havenith et al. [7] showed that relative spike timing in visual cortex reflects properties such as stimulus orientation. Importantly, given inter-connected pools of neurons, synchronous propagation of activity is more stable than asynchronous propagation. In fact, propagating synfire chains yield stable and robust spiking precision in the millisecond range that supports the self-stabilization of synfire chain activity [70]. That is, given the right starting conditions, initially weak synfire chains (with few active members or poor synchronization) can recruit additional members and reduce spike-timing variance across the group. However, slightly different initial conditions may force a synfire chain to cross a dynamical systems separatrix between attractors, forcing the synfire chain to quickly decay [71]. Since phase-to-rate mappings can influence spike timing, strong phase-to-rate mappings can increase the likelihood of some synfire chains while rendering others less likely. Since beta amplitude appears to act as a gain control mechanism for the strength of the phase-to-rate mapping, the influence of beta on the probability of different spike sequences can be adjusted by changing beta power. However, Figure 6 shows that a fixed change in beta amplitude will have a differential response on different cells, with some strongly increasing their phase preference while others show only moderate changes. Thus, the mapping from beta amplitude to spike sequence probabilities is not a simple one, but depends on the diversity of CLC parameters that hold across the population. Finally, task-dependent remapping of the preferred phase (c.f. Figures 5I,J) provides a mechanism for the selective and task-dependent control of synfire chain activation and propagation. That is, during a given task the relative probabilities of a set of (function-specific) multi-neuron spike sequences can be controlled via adjustments in beta amplitude, while switching to another task involves a remapping of CLC parameters in order to call a different set of spike sequences into action. Since motor cortical function involves both rate modulation as well as spike synchronization [72], [73], a mechanism to selective control synchronization while leaving rate modulation unchanged may prove useful to a system controlling distributed networks.

While the amplitude- and phase-to-rate mappings appear most relevant to local computation within a given cortical area, the phase-difference-to-rate mapping may play a role in the regulation of long-range communication between areas. According to the communication through coherence (CTC) hypothesis, the effective gain between interacting areas is a function of the phase difference between them [21], [51], [74]. It is difficult to see how the brain could implement CTC control systems without the use of neurons that detect phase differences between areas, on the one hand, as well as neurons than can evoke shifts in the relative phase between distal areas, on the other. Neurons that could serve as phase difference detectors and effectors appear to be fundamental elements required by any distributed system of oscillatory network control. Furthermore, hierarchical predictive coding models suggest that the gamma rhythm is indicative of bottom-up feed-forward processing, while the alpha and beta rhythms serve as signatures of top-down feedback influence [75]–[78]. Distinct phase-difference-to-rate mappings that operate at these frequencies appear to be one way to control the relative balance of feedforward and feedback processing. The phase-difference to amplitude-envelope-correlation relationship shown in Figure S8D appears to support the communication through coherence hypothesis, but further studies targeting the role of spiking neurons in long-range interactions are required to clarify their role in the oscillatory control of distributed networks.

Prior work studying neural dynamics in motor cortex has tended to focus on the correlation between spiking activity and “external” factors (e.g. movement velocity, environmental state, behavior-dependent sensory feedback, etc). In contrast, this study focused on “internal” factors that arise from spontaneous, ongoing brain activity – including beta amplitude and phase within an area, or the difference in beta phase between areas. Specifically, we showed that most neurons exhibited a sigmoid dependence on beta amplitude (considered alone; Figure 4), a cosine dependence on beta phase (considered alone; Figure 5), and that beta amplitude provided a quadratic gain control for the beta phase preference (Figure 6). What is the relationship between these “external” and “internal” factors? Figure 3 provides an example of external and internal tuning for one example neuron, showing how input variables can be mapped to a predicted rate, which can then be compared to a measured rate. For example, Figure 3D shows how time-in-trial and target ID can be mapped to a predicted rate (color), while Figure 3E shows how this predicted rate compares to the rate that actually occurs. Similarly, Figure 3I–J show this for beta amplitude and phase; these figures show that the range of the predicted rate generated from the beta-to-rate mapping (r_internal_) is about half that of the range of the predicted rate generated from trial information (r_external_). The sum of these terms (r = r_external_ + r_internal_) often has a larger range than either r_external_ or r_internal_ alone. However, this sum assumes that r_external_ and r_internal_ are independent – an assumption that is not appropriate for many neurons. For example, while Figure 7G–H shows neurons where target direction appears independent of the phase-to-rate modulation depth, Figure 7E–F show examples where there is a clear interaction between internal and external factors. The focus of this study was to investigate the dependence of spiking on internal factors, and to determine if this dependence changes from one task to another. Determining the relation between internal and external factors will require further investigation.

Nonetheless, the majority of neurons show a dependence on “internal” beta-related factors that is not mediated by external factors such as direction tuning (Figure S3). A related concern is that the observed changes in CLC are more directly linked to bottom-up demands related to the trial substages (e.g. hold vs. movement period) than to top-down modulation associated with the task context. Figure S5 addresses this concern by directly comparing endogenous and exogenous factors; the fact that cross-task, within-stage differences are larger than within-task, cross-stage differences indicates that task context is a factor in determining neuronal responses related to CLC. That is, it appears that cells are influenced both by bottom-up, exogenous input related to the processing demands of the different trial substages as well as by top-down, endogenous input related to the maintenance of task context and rule selection.

An interesting aspect of this analysis has been the observation of the strong heterogeneity of neuronal sensitivities to different types of input, considering external vs. internal factors or top-down vs. bottom-up aspects of the experimental demands, compared to the stability of the average population responses. For example, Figure S3A shows the baseline firing rates for each neuron during BC and MC, and makes it clear that many neurons exhibit large task-dependent shifts in the baseline spike rate. The average spike rate over the population, however, is relatively unchanged (red and blue lines, Figure S3A). That is, with a shift in task the neuronal ensemble seems to reassign firing rates around a constant population mean rate. Similarly, the amplitude-to-rate and phase-to-rate mappings computed using spikes from all neurons (average population mappings) do not show the strong task-dependent shifts seen in the mappings of individual neurons. Therefore, we would predict that electrophysiological measures that depend on average ensemble activity, such as coupling between beta and the broadband ECoG signal [79], will be less likely to exhibit strong task-dependent changes than will individual neurons.

Finally, the empirical findings reported here are consistent with the hypothesis that dynamic changes in coupling between multiple spatial and temporal scales provide a simple mechanism to bias functional network activity [80]. In particular, coupling between single neurons and the motor beta rhythm exhibits several properties that appear positioned to influence local cortical computation – namely, the phase-regulation of relative spike timing on a scale of tens of milliseconds and the amplitude-regulation of winner-take-all dynamics within neuronal ensembles occurring on a scale of hundreds of milliseconds. Similarly, long-distance communication appears to be modulated by the relative phase difference between areas. The presence of neurons that are sensitive to these properties could provide a mechanistic route for this information about relative phase differences to be detected and actively used in the dynamic regulation of large-scale network activity. While future studies employing casual intervention will be required to fully test the functional role of different oscillatory rhythms, here we have shown that the mapping from beta activity to firing rate changes in a reversible, task-dependent way. Given that beta oscillations are generated by the coordinated population activity of hundreds of thousand cells involved in a distributed network that spans both hemispheres [11], the results presented here suggests that the relationship of multiscale coupling between single neurons and larger networks is flexible and can be dynamically remapped in order to support new functional roles.

## Supporting Information

Figure S1 **Task-dependent changes in the beta amplitude-to-rate and phase-to-rate mappings for Monkey R.** A–F) As in Figure 4B–G, for monkey R. Shown are six example neurons indicating the range of within-neuron remapping that may occur when moving from BC (red) to MC (blue). G–L) As in Figure 5A–H, for monkey R. Six example neurons from left primary motor cortex (M1) showing task-dependent remapping of the beta phase-to-rate relationship.
(TIF)

Figure S2 **Amplitude-to-rate mapping: within-task mapping stability and cross-task remapping reliability.** A–B) Colors and neurons are as in Figure 4A, but the BC data has been spilt into 2 disjoint dataset prior to computing the amplitude-to-rate mappings. For each pair of neurons, if the amplitude-to-rate mappings for the pair cross, then the rank order of that pair (in terms of spike rate associated with beta amplitude alone) also switches (c.f. Figure 11). The beta amplitude value corresponding to such rank-order switches is stable within task but can change across tasks. More generally, amplitude-to-rate mappings exhibit within-task stability and cross-task diversity. C) For each cell, plotting the within-task amplitude-to-rate mappings against each other reveals few changes; amplitude-to-rate mapping for BC1 versus the amplitude-to-rate mapping for BC2.. D–E) As in A–B, for the MC task. F) As in C, for the MC task. G) In contrast to these within-task comparisons, plotting the amplitude-to-rate mappings for a given cell across tasks reveals large but reproducible changes; BC1 versus MC1 shows that neurons can exhibit task-dependent changes. H) As in G, for BC2 vesus MC2. I) For any given amplitude, the amplitude-to-rate mappings for the ensemble induce a rank ordering in terms of spike rate (i.e., the spike rate rank ordering that would hold if cells received no input other than that associated with the amplitude-to-rate mapping). For the 12 example neurons shown here, 41 distinguishable rank orderings occur across BC1, BC2, MC1, and MC2. Each rank order can be named with an (arbitrary) rank-order state ID, and each rank order state may have different neurocomputational consequences due to winner-take-all dynamics within an ensemble (c.f. Figure 11). Importantly, stable within-task amplitude-to-rate mappings for an ensemble establishes a sequence of rank order states; smoothly moving from low to high amplitude will correspond to a specific sequence of discrete rank-order states. That is, for each task there is a mapping from the one-dimensional amplitude axis to an ordered sequence of discrete ensemble states (while the state ID is arbitrary, the sequence of states is determined by the task, and each state is index by a fixed interval of beta amplitude values). Switching tasks leads to a remapping of amplitude-to-rate mappings across the ensemble, which results in amplitude being mapped to different sequence of rank-order states. Shown are the rank-order state IDs associated with each beta amplitude during BC1 (solid red line), BC2 (dashed red), MC1 (solid blue), and MC2 (dashed blue); the ensemble rate-based sequence ordering indexed by beta amplitude is preserved within tasks, but may change between tasks.
(TIF)

Figure S3 **The relation of internal and external tuning properties.** Relation of amplitude- and phase-to-rate mapping parameters to baseline rate, direction tuning modulation depth, and direction tuning preferred direction. A) Baseline spike rates in BC and MC conditions. Average ensemble rate is approximately the same in BC (red line) and MC (blue line). B) Range of rate change for amplitude-to-rate mapping vs. baseline rate in BC (red) and MC (blue). Open circles (asterisks) indicate mappings with negative (positive) correlation between beta amplitude and spike rate. C) Range of rate change for phase-to-rate mapping vs. baseline rate in BC (red) and MC (blue). D) Phase-to-rate mapping preferred angle vs. baseline rate in BC (red) and MC (blue). E) Target-specific direction tuning modulation depth in BC and MC. F) As in B, but showing the relation of amplitude-to-rate spike rate range vs. direction tuning modulation depth rather than baseline rate. G) Range of rate change for phase-to-rate mapping vs. direction-tuning modulation depth. H) Phase-to-rate preferred beta angle vs. direction tuning modulation depth. I) Preferred movement direction in BC and MC. J) As in B, but showing the relation of spike rate range vs. preferred movement direction. K) Phase-to-rate spike rate range vs. preferred direction. L) Phase-to-rate preferred beta angle vs. preferred movement direction.
(TIF)

Figure S4 **Phase-to-rate mapping: within-task mapping stability and cross-task remapping reliability.** The phase-to-rate mapping was computed separately for 4 datasets: 2 BC blocks (BC1 and BC2) and 2 MC blocks (MC1 and MC2). Both the modulation depth (upper right) and preferred beta angle (lower left) are more similar for within-task comparisons (BC1/BC2, red; MC1/MC2, blue) than for cross-task comparisons (BC1/MC1, BC1/MC2, BC2/MC1, BC2/MC2, black). While the parameters for a given neuron differ from BC to MC, task-dependent changes are reproducible across disjoint datasets (e.g., compare modulation depth scatterplots for BC1/MC1 to BC2/MC2).
(TIF)

Figure S5 **Task- and trial-substage dependence of amplitude-to-rate mapping.** The amplitude-to-rate mapping was computed separately for 8 datasets forming a 2 × 2 × 2 set distinguishing task (MC or BC), trial sub-stage (Move-to-Center or Move-to-Target), and disjoint group (1 for odd trials or 2 or even trials). Parameter comparisons across datasets highlight different contrasts, including: 1) within-task, within-trial-stage (wTwS); 2) within-task, cross-trial-stage (wTcS); and 3) cross-task, within-trial-stage (cTwS). Task-dependent changes in the linear slope of the amplitude-to-rate mapping are isolated by cTwS, while trial-stage-dependent changes are isolated by the wTcS comparison.
(TIF)

Figure S6 **Task- and trial-substage dependence of phase-to-rate mapping modulation depth.** The phase-to-rate mapping was computed separately for 8 datasets forming a 2 × 2 × 2 set distinguishing task (MC or BC), trial sub-stage (Move-to-Center or Move-to-Target), and disjoint group (1 for odd trials or 2 or even trials). Parameter comparisons across datasets highlight different contrasts, including: 1) within-task, within-trial-stage (wTwS); 2) within-task, cross-trial-stage (wTcS); and 3) cross-task, within-trial-stage (cTwS). Task-dependent changes in phase-to-rate modulation depth are isolated by cTwS, while trial-stage-dependent changes are isolated by the wTcS comparison.
(TIF)

Figure S7 **Task- and trial-substage dependence of phase-to-rate mapping preferred angle.** The phase-to-rate mapping was computed separately for 8 datasets forming a 2 × 2 × 2 set distinguishing task (MC or BC), trial sub-stage (Move-to-Center or Move-to-Target), and disjoint group (1 for odd trials or 2 or even trials). Parameter comparisons across datasets highlight different contrasts, including: 1) within-task, within-trial-stage (wTwS); 2) within-task, cross-trial-stage (wTcS); and 3) cross-task, within-trial-stage (cTwS). Task-dependent changes in phase-to-rate preferred angle are isolated by cTwS, while trial-stage-dependent changes are isolated by the wTcS comparison.
(TIF)

Figure S8 **Mapping from inter-hemispheric phase difference to mean beta amplitude and inter-hemispheric amplitude envelope correlations.** A) Mean beta amplitude is statistically dependent on the beta phase difference between left M1 (M1l) and right M1 (M1r) during both MC and BC. However, the optimal lag between the amplitude and phase difference time series depends on task condition. During BC, the optimal lag for M1l and M1r is 0 ms and +1 ms, respectively, but during MC the optimal lag shifts to −6 ms for M1l and +10 ms for M1r. M1l drives movement of the right arm during MC; one possibility is that M1l amplitude changes precede changes in the inter-hemispheric M1l/M1r phase difference (6 ms later), while the M1l/M1r phase difference best predicts M1r amplitudes 10 ms later. B) Mean beta amplitude as a function of the inter-hemispheric beta phase difference, shown for optimal lags (c.f. Figure S8A). Amplitudes are normalized to a mean of 1 for all data; differences from unity imply that the beta amplitude distribution changes when conditioned on the inter-hemispheric phase difference. C) The range of inter-hemispheric amplitude correlations conditioned on the inter-hemispheric phase difference for a set of time lags. In both MC and BC, the phase difference is most predictive of amplitude correlations occurring 26 ms later. The origin of the double peak symmetric around lag 0 is unclear. D) The mapping between inter-hemispheric beta phase differences to beta amplitude correlations, plotted for the optimal lag (+26 ms). This mapping implies that knowing the phase difference between left and right M1 provides knowledge about the amplitude correlations (between M1l and M1r) that will hold 26 ms later.
(TIF)

Figure S9 **Amplitude-to-rate mapping exhibits independent task- and trial-stage related changes.** A–I) Amplitude-to-rate mapping for three example neurons (sig086a, top row; sig099a, middle row; sig104a, bottom row) over three trial sub-stages (Move-to-Center, left column; Move-to-Target, middle column; Reward On, right column) showing that trial-stage differences do not account for task-related differences.
(TIF)

Figure S10 **Baseline-corrected amplitude-to-rate mappings for seven neurons.** The change in firing rate as a function of beta amplitude for seven example neurons: sig015a (blue), sig029a (green), sig029b (red), sig031a (cyan), sig045a (purple), sig062a (gold), and sig081b (black). Baseline rate has been removed to emphasize rate changes associated with amplitude variation.
(TIF)

Table S1 **The fraction of neurons showing significant changes in spike rate as a function of beta amplitude (the amplitude-to-rate mapping).** Percentages were computed separately for Monkeys P and R during the BC and MC tasks (columns), and stratified by correlation type – positive, negative, or no correlation (rows).
(DOCX)

Table S2 **The fraction of neurons showing task-dependent changes in the beta amplitude to rate mapping, stratified by correlation direction (positive or negative) and relative strength of modulation during the MC and BC tasks (rows), computed separately for Monkeys P and R.**
(DOCX)

Table S3 **The fraction of neurons exhibiting changes in spike rate as a function of beta phase, computed separately for Monkeys P and R (columns) over the MC and BC tasks (rows).**
(DOCX)
