# Supplementary figures and images for: Task-Dependent Changes in Cross-Level Coupling between Single Neurons and Oscillatory Activity in Multiscale Networks
Source: PLoS Comput Biol. 2012 Dec 20;8(12):e1002809. doi: 10.1371/journal.pcbi.1002809 (PMC3527280; doi:10.1371/journal.pcbi.1002809)

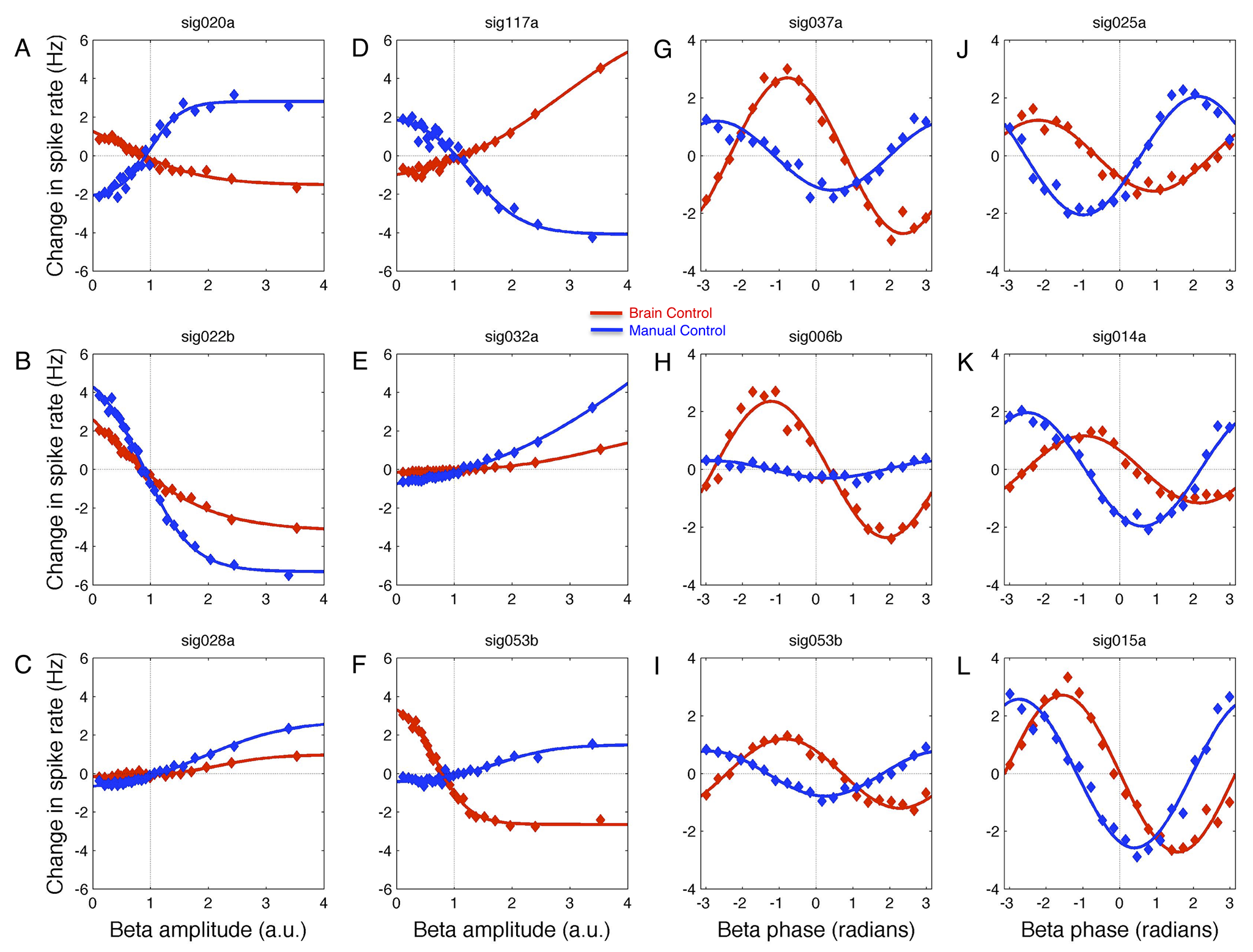

Supplement: Figure S1 — Task-dependent changes in the beta amplitude-to-rate and phase-to-rate mappings for Monkey R. A–F) As in Figure 4B–G, for monkey R. Shown are six example neurons indicating the range of within-neuron remapping that may occur when moving from BC (red) to MC (blue). G–L) As in Figure 5A–H, for monkey R. Six example neurons from left primary motor cortex (M1) showing task-dependent remapping of the beta phase-to-rate relationship. (TIF) [file pcbi.1002809.s001.tif]

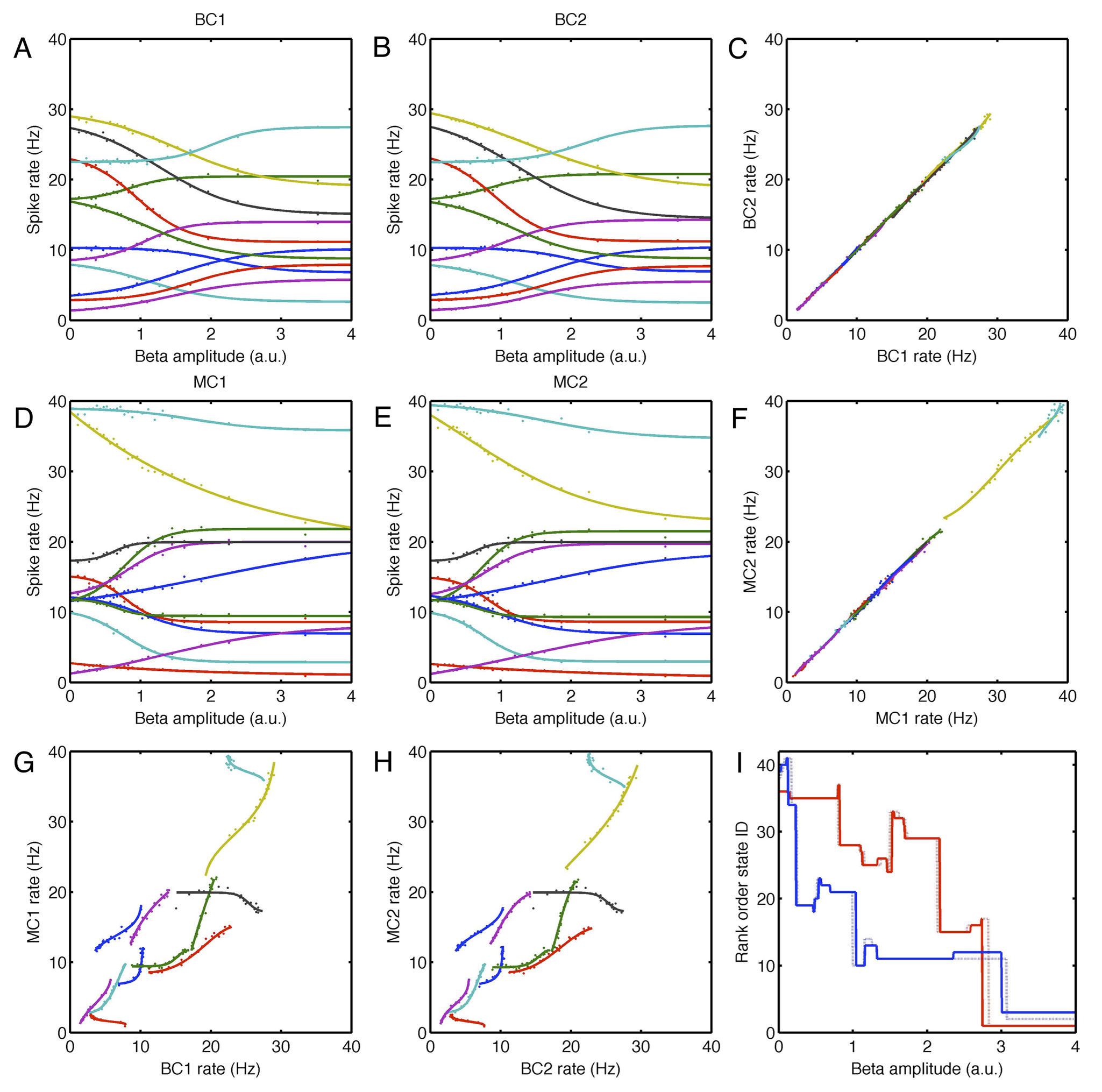

Supplement: Figure S2 — Amplitude-to-rate mapping: within-task mapping stability and cross-task remapping reliability. A–B) Colors and neurons are as in Figure 4A, but the BC data has been spilt into 2 disjoint dataset prior to computing the amplitude-to-rate mappings. For each pair of neurons, if the amplitude-to-rate mappings for the pair cross, then the rank order of that pair (in terms of spike rate associated with beta amplitude alone) also switches (c.f. Figure 11). The beta amplitude value corresponding to such rank-order switches is stable within task but can change across tasks. More generally, amplitude-to-rate mappings exhibit within-task stability and cross-task diversity. C) For each cell, plotting the within-task amplitude-to-rate mappings against each other reveals few changes; amplitude-to-rate mapping for BC1 versus the amplitude-to-rate mapping for BC2.. D–E) As in A–B, for the MC task. F) As in C, for the MC task. G) In contrast to these within-task comparisons, plotting the amplitude-to-rate mappings for a given cell across tasks reveals large but reproducible changes; BC1 versus MC1 shows that neurons can exhibit task-dependent changes. H) As in G, for BC2 vesus MC2. I) For any given amplitude, the amplitude-to-rate mappings for the ensemble induce a rank ordering in terms of spike rate (i.e., the spike rate rank ordering that would hold if cells received no input other than that associated with the amplitude-to-rate mapping). For the 12 example neurons shown here, 41 distinguishable rank orderings occur across BC1, BC2, MC1, and MC2. Each rank order can be named with an (arbitrary) rank-order state ID, and each rank order state may have different neurocomputational consequences due to winner-take-all dynamics within an ensemble (c.f. Figure 11). Importantly, stable within-task amplitude-to-rate mappings for an ensemble establishes a sequence of rank order states; smoothly moving from low to high amplitude will correspond to a specific sequence of discrete rank-order s [file pcbi.1002809.s002.tif]

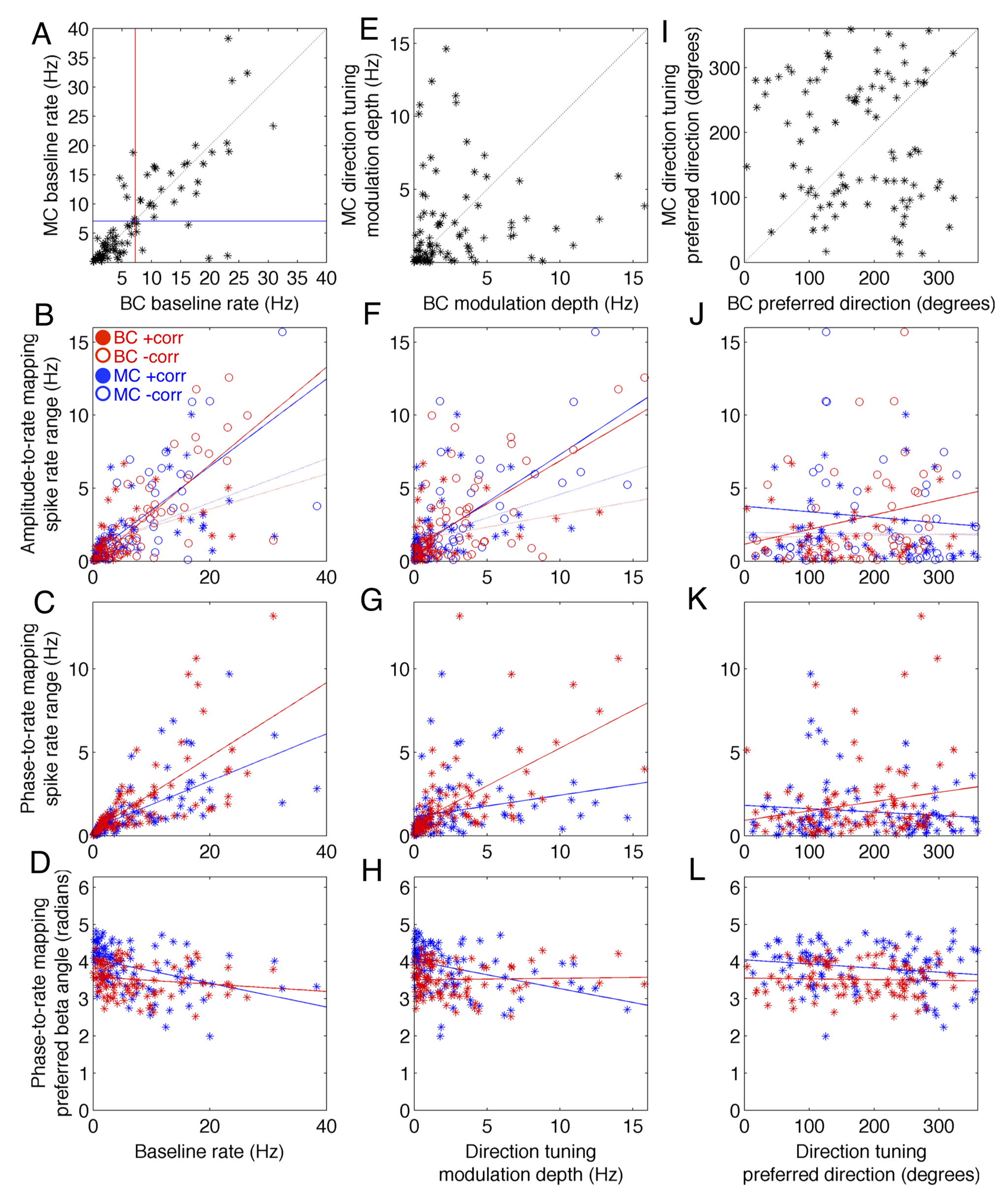

Supplement: Figure S3 — The relation of internal and external tuning properties. Relation of amplitude- and phase-to-rate mapping parameters to baseline rate, direction tuning modulation depth, and direction tuning preferred direction. A) Baseline spike rates in BC and MC conditions. Average ensemble rate is approximately the same in BC (red line) and MC (blue line). B) Range of rate change for amplitude-to-rate mapping vs. baseline rate in BC (red) and MC (blue). Open circles (asterisks) indicate mappings with negative (positive) correlation between beta amplitude and spike rate. C) Range of rate change for phase-to-rate mapping vs. baseline rate in BC (red) and MC (blue). D) Phase-to-rate mapping preferred angle vs. baseline rate in BC (red) and MC (blue). E) Target-specific direction tuning modulation depth in BC and MC. F) As in B, but showing the relation of amplitude-to-rate spike rate range vs. direction tuning modulation depth rather than baseline rate. G) Range of rate change for phase-to-rate mapping vs. direction-tuning modulation depth. H) Phase-to-rate preferred beta angle vs. direction tuning modulation depth. I) Preferred movement direction in BC and MC. J) As in B, but showing the relation of spike rate range vs. preferred movement direction. K) Phase-to-rate spike rate range vs. preferred direction. L) Phase-to-rate preferred beta angle vs. preferred movement direction. (TIF) [file pcbi.1002809.s003.tif]

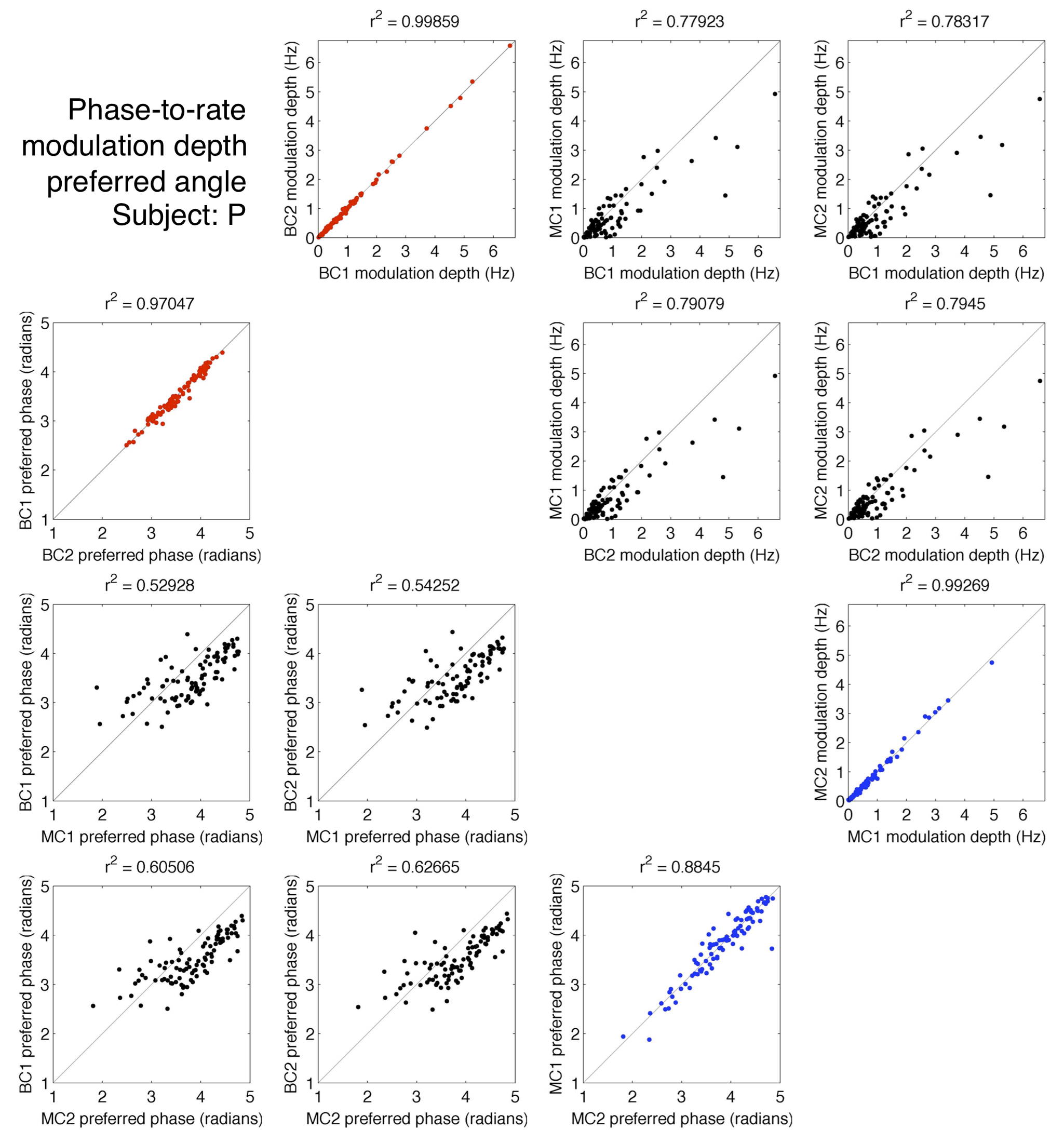

Supplement: Figure S4 — Phase-to-rate mapping: within-task mapping stability and cross-task remapping reliability. The phase-to-rate mapping was computed separately for 4 datasets: 2 BC blocks (BC1 and BC2) and 2 MC blocks (MC1 and MC2). Both the modulation depth (upper right) and preferred beta angle (lower left) are more similar for within-task comparisons (BC1/BC2, red; MC1/MC2, blue) than for cross-task comparisons (BC1/MC1, BC1/MC2, BC2/MC1, BC2/MC2, black). While the parameters for a given neuron differ from BC to MC, task-dependent changes are reproducible across disjoint datasets (e.g., compare modulation depth scatterplots for BC1/MC1 to BC2/MC2). (TIF) [file pcbi.1002809.s004.tif]

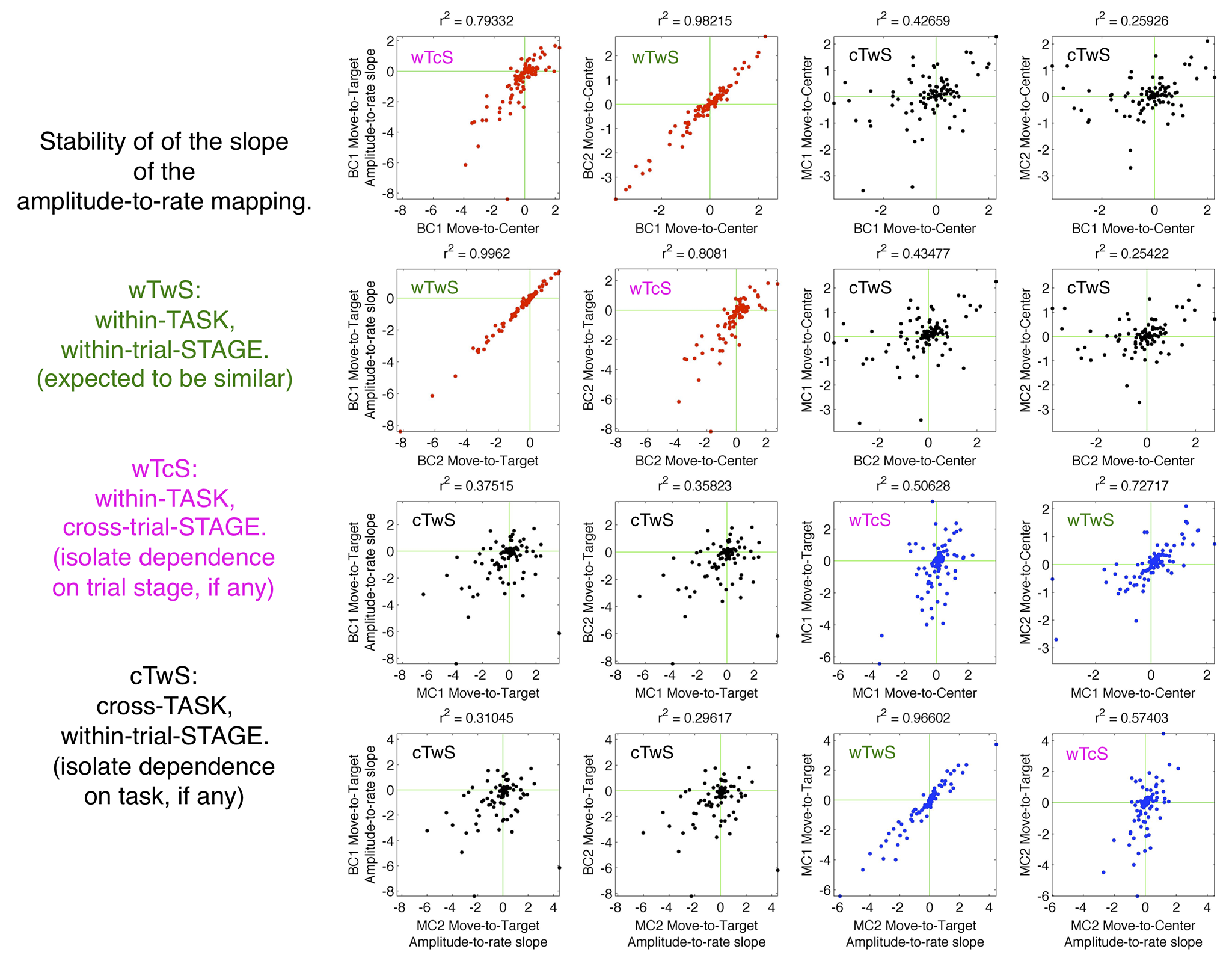

Supplement: Figure S5 — Task- and trial-substage dependence of amplitude-to-rate mapping. The amplitude-to-rate mapping was computed separately for 8 datasets forming a 2 × 2 × 2 set distinguishing task (MC or BC), trial sub-stage (Move-to-Center or Move-to-Target), and disjoint group (1 for odd trials or 2 or even trials). Parameter comparisons across datasets highlight different contrasts, including: 1) within-task, within-trial-stage (wTwS); 2) within-task, cross-trial-stage (wTcS); and 3) cross-task, within-trial-stage (cTwS). Task-dependent changes in the linear slope of the amplitude-to-rate mapping are isolated by cTwS, while trial-stage-dependent changes are isolated by the wTcS comparison. (TIF) [file pcbi.1002809.s005.tif]

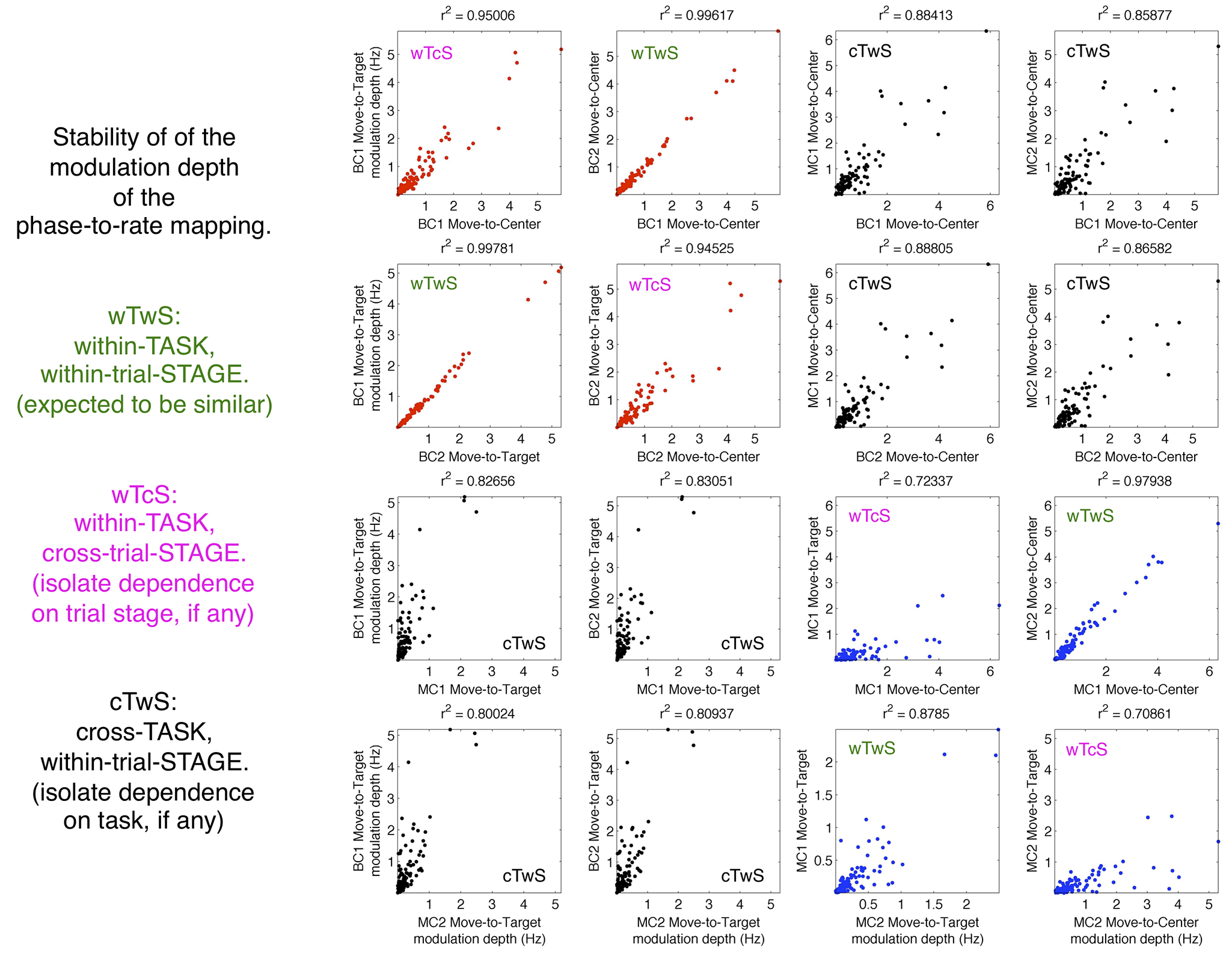

Supplement: Figure S6 — Task- and trial-substage dependence of phase-to-rate mapping modulation depth. The phase-to-rate mapping was computed separately for 8 datasets forming a 2 × 2 × 2 set distinguishing task (MC or BC), trial sub-stage (Move-to-Center or Move-to-Target), and disjoint group (1 for odd trials or 2 or even trials). Parameter comparisons across datasets highlight different contrasts, including: 1) within-task, within-trial-stage (wTwS); 2) within-task, cross-trial-stage (wTcS); and 3) cross-task, within-trial-stage (cTwS). Task-dependent changes in phase-to-rate modulation depth are isolated by cTwS, while trial-stage-dependent changes are isolated by the wTcS comparison. (TIF) [file pcbi.1002809.s006.tif]

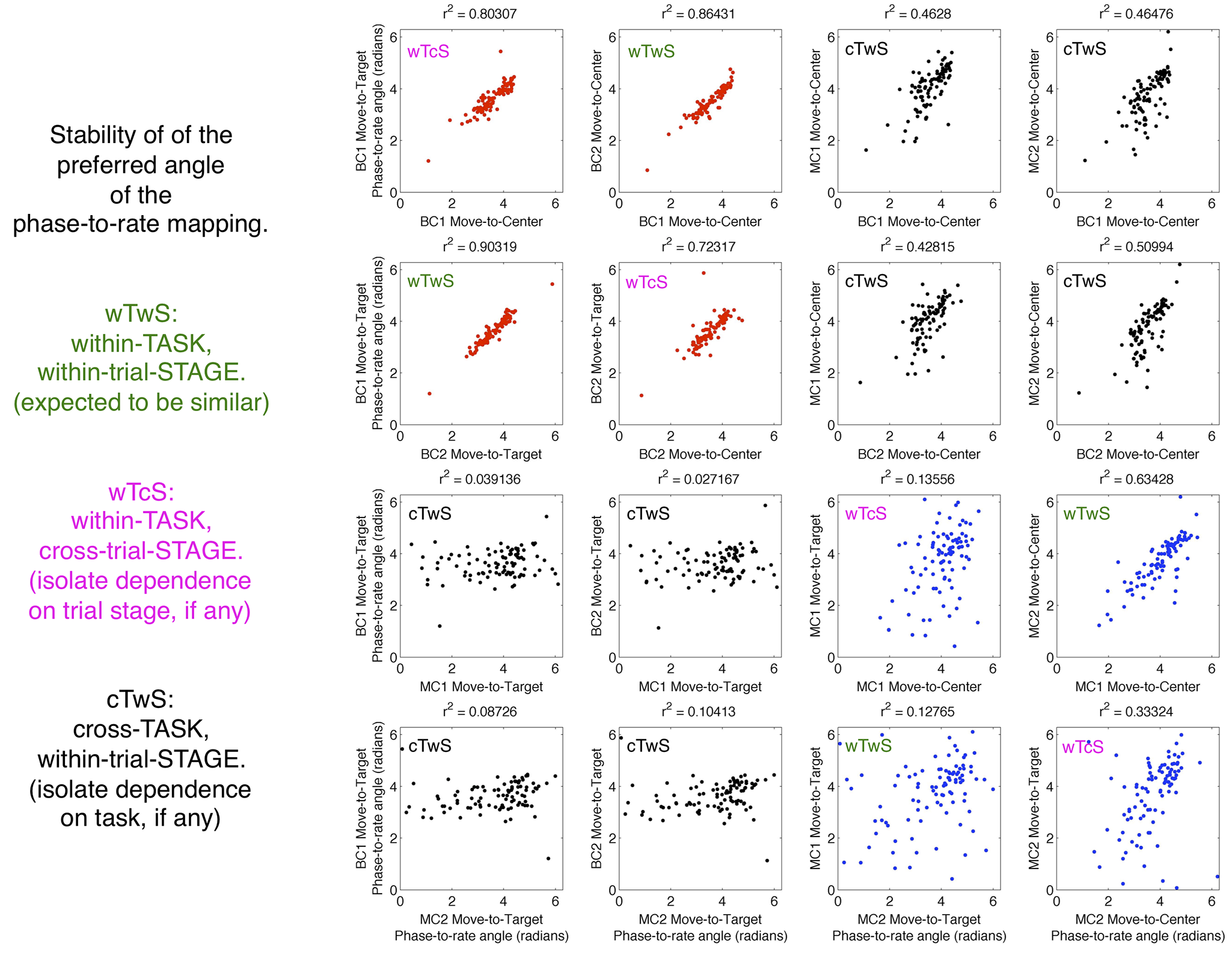

Supplement: Figure S7 — Task- and trial-substage dependence of phase-to-rate mapping preferred angle. The phase-to-rate mapping was computed separately for 8 datasets forming a 2 × 2 × 2 set distinguishing task (MC or BC), trial sub-stage (Move-to-Center or Move-to-Target), and disjoint group (1 for odd trials or 2 or even trials). Parameter comparisons across datasets highlight different contrasts, including: 1) within-task, within-trial-stage (wTwS); 2) within-task, cross-trial-stage (wTcS); and 3) cross-task, within-trial-stage (cTwS). Task-dependent changes in phase-to-rate preferred angle are isolated by cTwS, while trial-stage-dependent changes are isolated by the wTcS comparison. (TIF) [file pcbi.1002809.s007.tif]

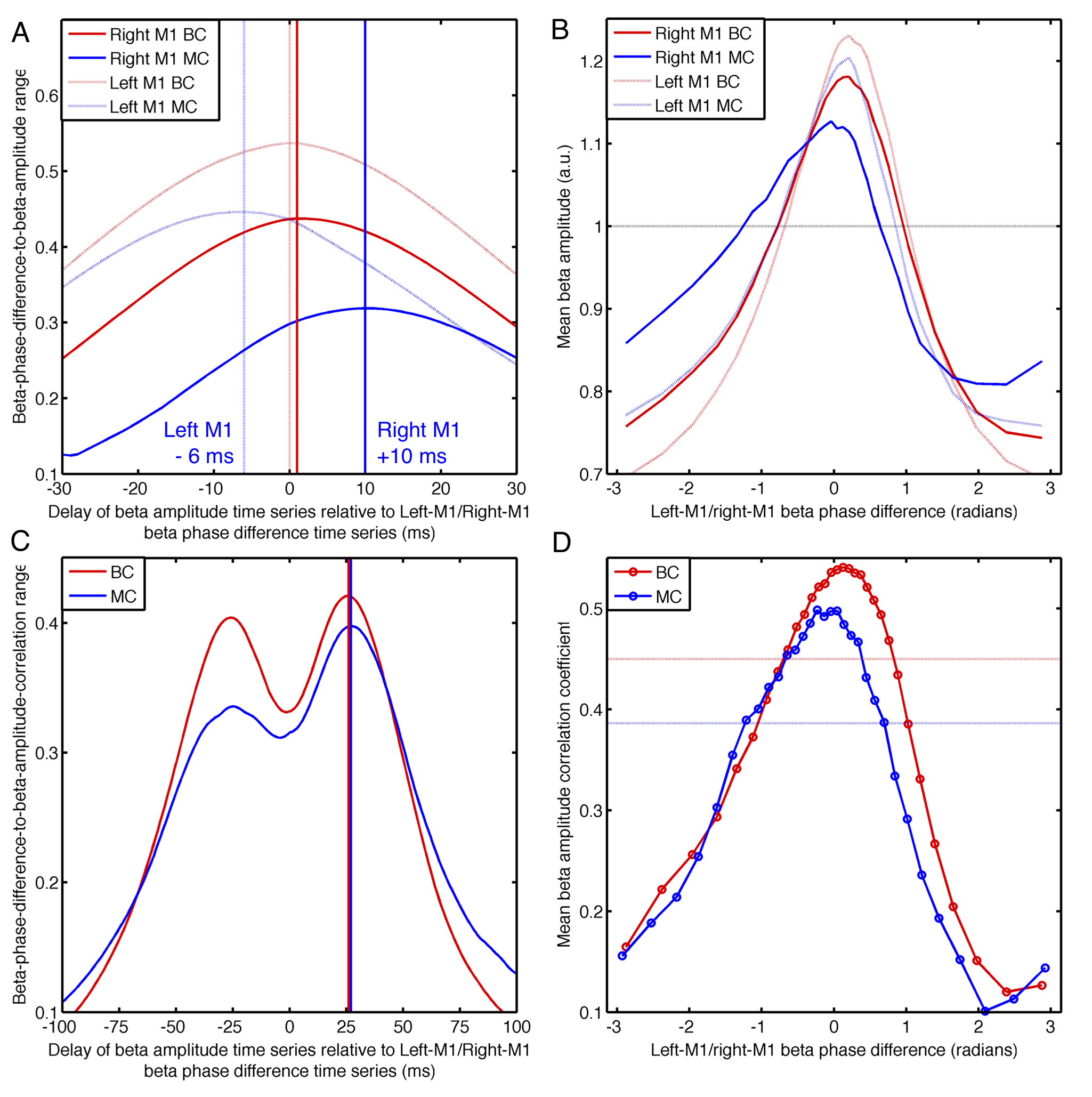

Supplement: Figure S8 — Mapping from inter-hemispheric phase difference to mean beta amplitude and inter-hemispheric amplitude envelope correlations. A) Mean beta amplitude is statistically dependent on the beta phase difference between left M1 (M1l) and right M1 (M1r) during both MC and BC. However, the optimal lag between the amplitude and phase difference time series depends on task condition. During BC, the optimal lag for M1l and M1r is 0 ms and +1 ms, respectively, but during MC the optimal lag shifts to −6 ms for M1l and +10 ms for M1r. M1l drives movement of the right arm during MC; one possibility is that M1l amplitude changes precede changes in the inter-hemispheric M1l/M1r phase difference (6 ms later), while the M1l/M1r phase difference best predicts M1r amplitudes 10 ms later. B) Mean beta amplitude as a function of the inter-hemispheric beta phase difference, shown for optimal lags (c.f. Figure S8A). Amplitudes are normalized to a mean of 1 for all data; differences from unity imply that the beta amplitude distribution changes when conditioned on the inter-hemispheric phase difference. C) The range of inter-hemispheric amplitude correlations conditioned on the inter-hemispheric phase difference for a set of time lags. In both MC and BC, the phase difference is most predictive of amplitude correlations occurring 26 ms later. The origin of the double peak symmetric around lag 0 is unclear. D) The mapping between inter-hemispheric beta phase differences to beta amplitude correlations, plotted for the optimal lag (+26 ms). This mapping implies that knowing the phase difference between left and right M1 provides knowledge about the amplitude correlations (between M1l and M1r) that will hold 26 ms later. (TIF) [file pcbi.1002809.s008.tif]

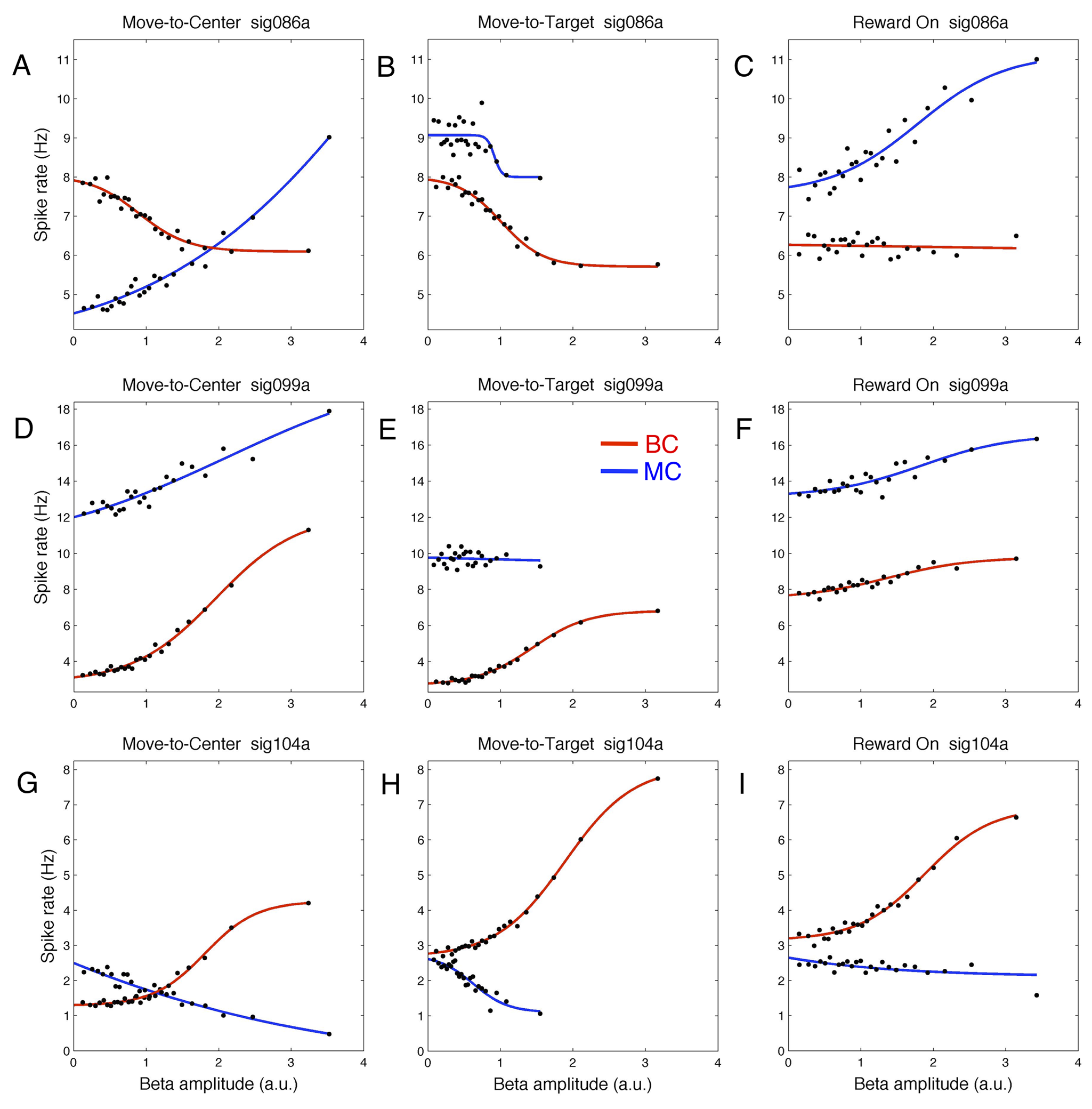

Supplement: Figure S9 — Amplitude-to-rate mapping exhibits independent task- and trial-stage related changes. A–I) Amplitude-to-rate mapping for three example neurons (sig086a, top row; sig099a, middle row; sig104a, bottom row) over three trial sub-stages (Move-to-Center, left column; Move-to-Target, middle column; Reward On, right column) showing that trial-stage differences do not account for task-related differences. (TIF) [file pcbi.1002809.s009.tif]

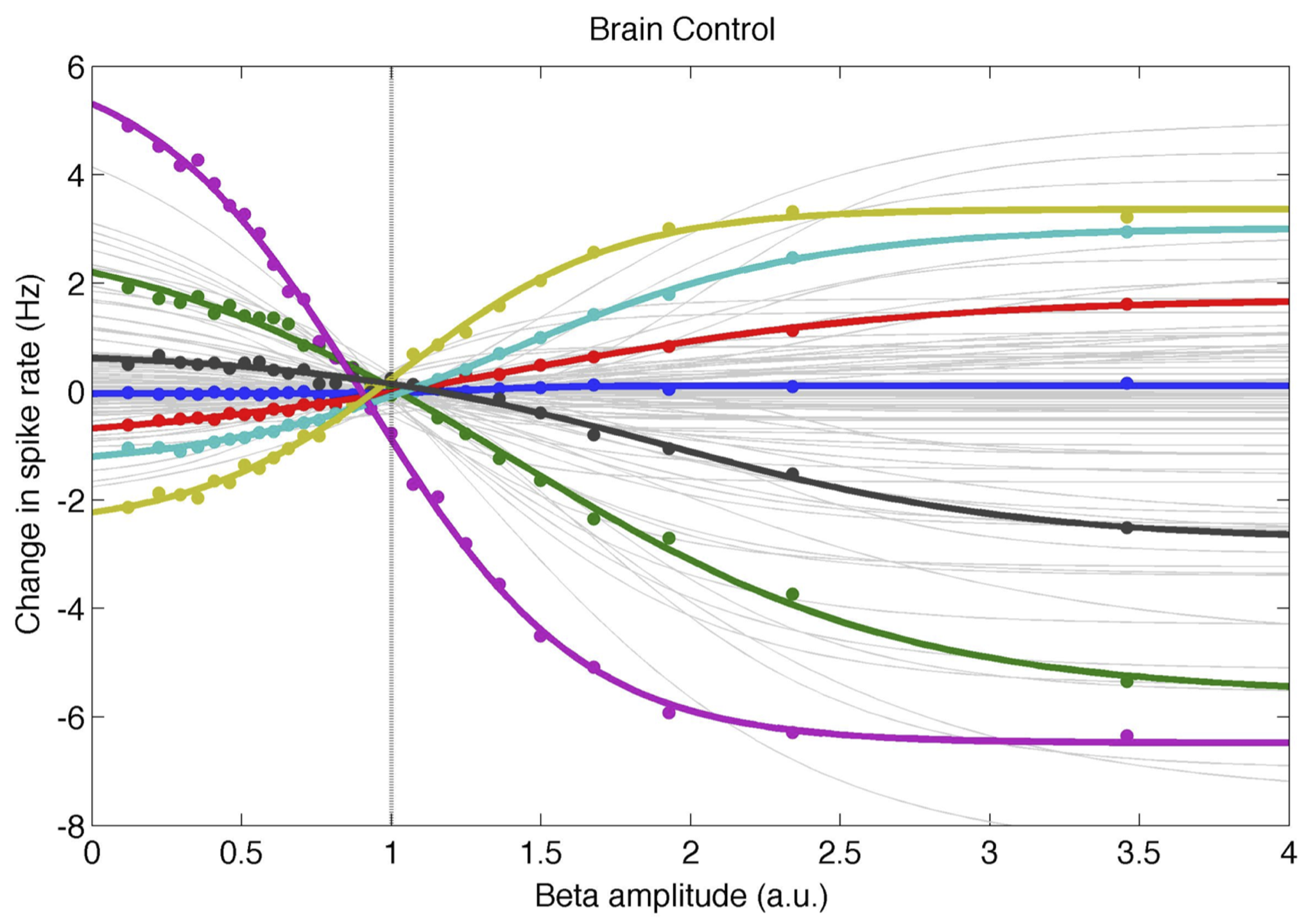

Supplement: Figure S10 — Baseline-corrected amplitude-to-rate mappings for seven neurons. The change in firing rate as a function of beta amplitude for seven example neurons: sig015a (blue), sig029a (green), sig029b (red), sig031a (cyan), sig045a (purple), sig062a (gold), and sig081b (black). Baseline rate has been removed to emphasize rate changes associated with amplitude variation. (TIF) [file pcbi.1002809.s010.tif]
