# Supplementary material for: Task-Dependent Changes in Cross-Level Coupling between Single Neurons and Oscillatory Activity in Multiscale Networks
Source: PLoS Comput Biol. 2012 Dec 20;8(12):e1002809. doi: 10.1371/journal.pcbi.1002809 (PMC3527280; doi:10.1371/journal.pcbi.1002809)
Supplement: Table S1 — The fraction of neurons showing significant changes in spike rate as a function of beta amplitude (the amplitude-to-rate mapping). Percentages were computed separately for Monkeys P and R during the BC and MC tasks (columns), and stratified by correlation type – positive, negative, or no correlation (rows). (DOCX) [file pcbi.1002809.s011.docx]

Table S1: The beta amplitude-to-rate mapping

| Table 1 | Monkey P  BC task | Monkey P  MC task | Monkey R  BC task | Monkey R  MC task | Combined  BC task | Combined  MC task |
| --- | --- | --- | --- | --- | --- | --- |
| Spike rate ⇑ as  beta power ⇑  (+ correlation) | 41.1% (39/95) | 47.4% (45/95) | 14.0% (12/86) | 32.6% (28/86) | 28.2% (51/181) | 40.3% (73/181) |
| Spike rate ⇓ as  beta power ⇑  (- correlation) | 48.4% (46/95) | 31.6% (30/95) | 69.8% (60/86) | 39.5% (34/86) | 58.6% (106/181) | 35.4% (64/181) |
| No change as  beta power ⇑  (no correlation) | 10.5% (10/95) | 21.1% (20/95) | 16.3% (14/86) | 27.9% (24/86) | 13.3% (24/181) | 24.3% (44/181) |
