# Supplementary material for: Task-Dependent Changes in Cross-Level Coupling between Single Neurons and Oscillatory Activity in Multiscale Networks
Source: PLoS Comput Biol. 2012 Dec 20;8(12):e1002809. doi: 10.1371/journal.pcbi.1002809 (PMC3527280; doi:10.1371/journal.pcbi.1002809)
Supplement: Table S2 — The fraction of neurons showing task-dependent changes in the beta amplitude to rate mapping, stratified by correlation direction (positive or negative) and relative strength of modulation during the MC and BC tasks (rows), computed separately for Monkeys P and R. (DOCX) [file pcbi.1002809.s012.docx]

Table S2: Task dependent beta amplitude-to-rate remapping

| Table 2 | Monkey P | Monkey R | Combined |
| --- | --- | --- | --- |
| Positive correlation for both BC and MC,  BC modulation depth > MC modulation depth | 14.7% (10/68) | 6.3% (3/48) | 11.2% (13/116) |
| Positive correlation for both BC and MC,  BC modulation depth < MC modulation depth | 27.9% (19/68) | 4.2% (2/48) | 18.1% (21/116) |
| Negative correlation for both BC and MC,  BC modulation depth > MC modulation depth | 11.8% (8/68) | 29.2% (14/48) | 19.0% (22/116) |
| Negative correlation for both BC and MC,  BC modulation depth < MC modulation depth | 17.6% (12/68) | 33.3% (16/48) | 24.1% (28/116) |
| Positive correlation for BC, negative correlation for MC | 11.8% (8/68) | 12.5% (6/48) | 12.1% (14/116) |
| Negative correlation for BC, positive correlation for MC | 16.2% (11/68) | 14.6% (7/48) | 15.5% (18/116) |
