# Supplementary material for: Task-Dependent Changes in Cross-Level Coupling between Single Neurons and Oscillatory Activity in Multiscale Networks
Source: PLoS Comput Biol. 2012 Dec 20;8(12):e1002809. doi: 10.1371/journal.pcbi.1002809 (PMC3527280; doi:10.1371/journal.pcbi.1002809)
Supplement: Table S3 — The fraction of neurons exhibiting changes in spike rate as a function of beta phase, computed separately for Monkeys P and R (columns) over the MC and BC tasks (rows). (DOCX) [file pcbi.1002809.s013.docx]

Table S3: Beta phase-to-rate mapping

| Table 3 | Monkey P | Monkey R | Combined |
| --- | --- | --- | --- |
| BC task | 100.0% (95/95) | 73.3% (63/86) | 87.3% (158/181) |
| MC task | 95.8% (91/95) | 86.0% (74/86) | 91.2% (165/181) |
